# Love Thy Neighbour? Tropical Tree Growth and Its Response to Climate Anomalies Is Mediated by Neighbourhood Hierarchy and Dissimilarity in Carbon‐ and Water‐Related Traits

**DOI:** 10.1111/ele.70028

**Published:** 2025-04-08

**Authors:** Daniela Nemetschek, Claire Fortunel, Eric Marcon, Johanna Auer, Vincyane Badouard, Christopher Baraloto, Marion Boisseaux, Damien Bonal, Sabrina Coste, Elia Dardevet, Patrick Heuret, Peter Hietz, Sébastien Levionnois, Isabelle Maréchaux, Clément Stahl, Jason Vleminckx, Wolfgang Wanek, Camille Ziegler, Géraldine Derroire

**Affiliations:** ^1^ AMAP, Université de Montpellier, CIRAD, CNRS, INRAE, IRD Montpellier France; ^2^ UMR EcoFoG, AgroParisTech, CIRAD, CNRS, INRAE, Université des Antilles, Université de la Guyane Kourou France; ^3^ School of Biological Sciences University of Bristol Bristol UK; ^4^ Center of Microbiology and Environmental Systems Science University of Vienna Vienna Austria; ^5^ Department of Biological Sciences, Institute of Environment Florida International University Miami Florida USA; ^6^ INRAE Université de Lorraine, AgroParisTech, UMR SILVA Nancy France; ^7^ Institute of Botany BOKU University Vienna Austria; ^8^ Université Libre de Bruxelles Brussels Belgium; ^9^ University of Bordeaux, INRAE, UMR BIOGECO Pessac France; ^10^ Cirad UMR EcoFoG, AgroParisTech, CNRS, INRAE, Université des Antilles, Université de la Guyane, Kourou France; ^11^ Cirad, UPR Forêts et Sociétés University of Montpellier Montpellier France

**Keywords:** climate response, functional traits, neighbourhood interactions, plant–water relations, tree growth, tropical forests

## Abstract

Taxonomic diversity effects on forest productivity and response to climate extremes range from positive to negative, suggesting a key role for complex interactions among neighbouring trees. To elucidate how neutral interactions, hierarchical competition and resource partitioning between neighbours' shape tree growth and climate response in a highly diverse Amazonian forest, we combined 30 years of tree censuses with measurements of water‐ and carbon‐related traits. We modelled individual tree growth response to climate and neighbourhood to disentangle the relative effect of neighbourhood densities, trait hierarchies and dissimilarities. While neighbourhood densities consistently decreased growth, trait dissimilarity increased it, and both had the potential to influence climate response. Greater water conservatism provided a competitive advantage to focal trees in normal years, but water–spender neighbours reduced this effect in dry years. By underlining the importance of density and trait‐mediated neighbourhood interactions, our study offers a way towards improving predictions of forest dynamics.

## Graphical abstract

Combining 30 years of tree census data with measurements of water‐ and carbon‐related traits, our study elucidates how neutral interactions, hierarchical competition and resource partitioning between neighbours' shape tree growth and its response to climate anomalies in a highly diverse Amazonian forest. By underlining the importance of density and trait‐mediated neighbourhood interactions in shaping tropical tree growth and its response to climate, our study offers a way towards improving predictions of forest dynamics.

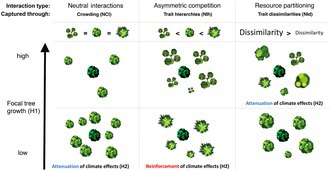

## 1 Introduction

Climate extremes such as heat waves, high atmospheric evaporative demands and low soil water availability (i.e., drought stress sensu lato), negatively affect forest productivity and functioning (Allen et al. 2010; Bauman, Fotunel, Cernusak, et al. 2022; Bauman, Fotunel, Delhaye, et al. 2022). These events are predicted to increase in frequency and intensity with ongoing climate change (Intergovernmental Panel on Climate Change [IPCC] 2023), which can alter global carbon dynamics (Higgins, Conradi, and Muhoko 2023). At the global scale, tree taxonomic diversity is an important driver of forest productivity (Liang et al. 2016), and can increase forest resistance to drought (Anderegg et al. 2018). However, at local scales such as individual forests or even tree neighbourhoods, the magnitude and even the sign of the effect of diversity on productivity can vary from site to site, depending on the local context (e.g., climate and disturbance regimes, stand structure and composition: Ammer 2019; Belote et al. 2011; Crawford et al. 2021) and temporal variations in resource availability or climate (Forrester and Bauhus 2016). Increasing evidence further suggests that diversity does not always increase forest resistance to droughts locally (Grossiord 2020; Pardos et al. 2021). Uncovering the mechanisms that underlie diversity effects on forest productivity and its response to climate is needed to better understand these context‐dependent effects (Grossiord 2020) and improve our ability to predict forest responses to climate change.

Complementarity in resource use among co‐occurring species has been proposed to explain increased forest productivity (Jucker, Bouriaud, Avacaritei, and Coomes 2014; Liang et al. 2015; Morin et al. 2011) and stability to environmental fluctuations, such as climate extremes (Loreau and de Mazancourt 2013; Schnabel et al. 2021) in species diverse stands. As competition for resources takes place at the neighbourhood scale, evidence for such an effect and its signature should be found in the influence of neighbours' identity on individual tree growth (Yu et al. 2024) and its response to climate. Neighbourhood species richness has been shown to influence individual functioning under various conditions (Fichtner et al. 2018, 2020). However, this taxonomic diversity lens only offers limited insights into the mechanisms that drive the effects of neighbourhood diversity, and especially whether complementarity actually plays a major role in the mitigation of negative climate effects (Grossiord 2020; Jucker, Bouriaud, Avacaritei, Dǎnilǎ, et al. 2014).

**FIGURE 1:**
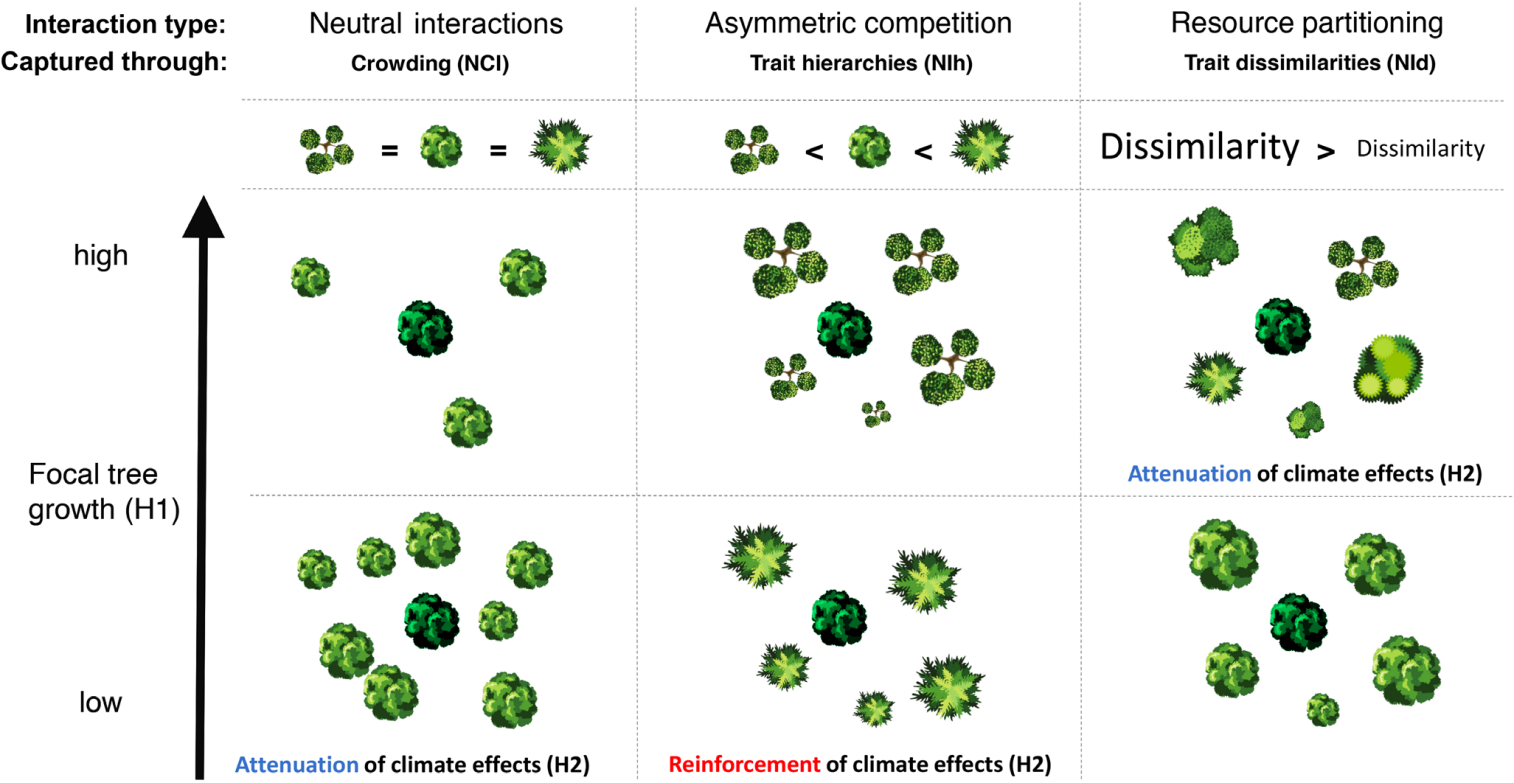
Conceptual illustration of different types of neighbourhood interactions, including neutral interactions, asymmetric competition and resource partitioning between a focal tree (central tree in dark green) and its neighbouring trees (light green). Neutral interactions can be captured by neighbourhood crowding indices (NCI), which depend solely on neighbourhood densities (i.e., the number, size and distance of neighbours). Asymmetric competition and resource partitioning can be respectively captured by the use of neighbourhood indices (NIh and NId) that include functional differences in the form of trait hierarchies (i.e., relative trait differences) or trait dissimilarities (i.e., absolute trait differences) between the focal tree and its neighbours. The expected effect of different types of neighbourhood interactions on individual tree growth (Hypothesis 1) and response to climate stress (Hypothesis 2), correspond to our hypotheses.

Neighbourhood effects on individual tree growth are the net outcome of simultaneous negative and positive biotic interactions between tree individuals in close proximity to each other (Begon, Townsend, and Harper 2006; Larocque et al. 2013). While their relative importance may change when heat and drought stress occur (Grossiord 2020), their net outcome can be captured by different neighbourhood indices (Figure 1). Negative neighbourhood effects can result from shared enemies or density‐dependent (i.e., neutral) interactions for shared resources (Jucker et al. 2016; Larocque et al. 2013; Pommerening and Sánchez Meador 2018). While denser neighbourhoods can reinforce drought effects (Bottero et al. 2017; Gleason et al. 2017), for instance through increased consumption of water, they can simultaneously shelter trees from atmospheric climate extremes (De Frenne et al. 2013; Nemetschek et al. 2024). Interactions between neighbouring trees from different species may additionally be asymmetric, suggesting that differences in species' functional strategies can result into competitive hierarchies between neighbours (Aschehoug et al. 2018; Canham, LePage, and Coates 2004; Craine and Dybzinski 2013; Pommerening and Sánchez Meador 2018). When water becomes limiting, dehydration‐tolerant species, which continue to transpire at low water potentials and species with high water demands, reflected by low water use efficiency and high transpiration rates (hereafter water spenders), likely exert higher pressure on shared water resources, than dehydration avoiding species or species with low water demands (hereafter water savers) (Delzon 2015; Martin‐StPaul, Delzon, and Cochard 2017; Volaire 2018). Therefore, water spender neigbours, may have greater negative impacts on drought stress experienced by water‐saver trees, than vice versa (Schnabel et al. 2024). Conversely, positive neighbourhood effects may result from facilitation (Brooker et al. 2007; Callaway 1995) or greater functional dissimilarity indicating resource partitioning (Hooper 1998; Pommerening and Sánchez Meador 2018), which could improve soil water availability for instance through hydraulic uplift and redistribution, therefore alleviating climate stress experienced by individual trees (Grossiord 2020; Hafner, Hesse, and Grams 2021; Prieto, Armas, and Pugnaire 2012). Previous work showed a key role of traits related to space, light and nutrients use in explaining the effect of trait difference dependent neighbourhood interactions on tree growth (Fortunel et al. 2016; Kunstler et al. 2016; Uriarte et al. 2010), but these traits offer little insights on water use strategies and responses to water limitations (Brodribb 2017; Grossiord 2020; Maréchaux et al. 2019; Wagner et al. 2014). In a previous study including traits related to light, nutrient and water use (Nemetschek et al. 2024), we showed that species traits of the focal tree alone partly captured interspecific differences in sensitivities to neighbourhood crowding and climate anomalies, but poorly captured their interactive effects. Examining how neighbourhood differences in water‐related traits influence neighbourhood interactions in normal and anomalous climate years, may therefore offer additional insight into neighbourhood interactions for water and elucidate their role in shaping individual response to drought (Grossiord 2020).

In the same highly diverse Amazonian forest in which we studied the effect of density‐dependent neighbourhood interactions and focal tree traits on species' climate response (Nemetschek et al. 2024), we now further investigate how neighbourhood trait differences in water‐related and carbon use traits shape tree growth and modulate its response to heat and drought stress. We used hierarchical Bayesian models to evaluate the separate and interactive effects of (i) climate variables indicating heat, atmospheric and soil water drought stress and (ii) neighbourhood indices capturing the effects of neighbourhood crowding, trait hierarchies and dissimilarities on individual tree growth. Our analyses leveraged trait and 30‐year long census data for 89 species from 15 permanent plots, some of which were subjected to initial selective logging and thinning, leading to contrasting neighbourhood structure, composition and dynamics. This long‐term census data and its high temporal resolution (biennial) provides a broad range of neighbourhood and climatic conditions (Figure S1) needed to study neighbourhood effects on climate responses of individual tree growth. We hypothesised that:

Hypothesis 1 *An individuals' growth is lower when surrounded by more neighbours (higher density), by superior competitors (stronger trait hierarchy) and more similar neighbours (lower trait dissimilarity)* (Table 1).

Hypothesis 2 *Neighbourhood is more likely to buffer negative climate effects when trait dissimilarity is high or when being composed of more water‐saver species which may positively contribute to overall water conservation at the neighbourhood scale. Conversely, high densities of water–spender and dehydration‐tolerant species are more likely to accentuate negative drought‐related climate effects* (Figure 1; Table 1).

**TABLE 1:** Functional traits used in the study, and their functional significance. We additionally provide the source from which data on each trait was compiled.

Organ	Trait	Abbreviation (Unit)	Function	Description	References	Data Source
**Traits pertaining to plant–water relations**						
Leaf	Bulk leaf carbon stable isotope	δ^13^C (‰)	Carbon–water use	High δ^13^C translates into high intrinsic water use efficiency (i.e., high photosynthetic rates relative to stomatal conductance) and therefore greater water conservation	Farquhar, Ehleringer, and Hubick (1989), Cernusak et al. (2013), Scheidegger et al. (2000)	Baraloto et al. (2010), Fortunel, Fine, and Baraloto (2012), Vleminckx et al. (2021)
Leaf	Water potential at turgor loss point	*π*_tlp_ (MPa)	Drought tolerance or water conservation	Low *π*_tlp_ translates into a greater ability to tolerate dehydration thereby maintaining stomatal conductance, hydraulic conductance and photosynthetic gas exchange at lower soil water potential. Conversely, high (i.e., less negative) π_tlp_ indicates early stomatal closure during drought, which allows avoiding dehydration through water conservation	Bartlett, Scoffoni, and Sack (2012), Martin‐StPaul, Delzon, and Cochard (2017)	Maréchaux et al. (2015, 2019), Nemetschek et al. (2024), Ziegler et al. (2019)
Leaf	Minimum conductance	*g*_min_ (mmol m^−2^ s^−1^)	Water conservation	Low *g*_min_ translates into low residual water loss after stomatal closure through leaf cuticle and incompletely closed stomata, thereby avoiding dehydration through water conservation	Blackman et al. (2019), Duursma et al. (2019), Machado et al. (2021)	Levionnois, Ziegler, et al. (2021), Nemetschek et al. (2024)
Leaf	Leaf saturated water content	LSWC (%)	Water storage	High LSWC translates into leaf water reserves that may allow maintenance of leaf water potential when water supply becomes limited	Blackman et al. (2019), Gleason et al. (2014), Luo et al. (2021)	Nemetschek et al. (2024)
**Traits pertaining to carbon use**						
Leaf	Leaf area	LA (cm^2^)	Light capture	Larger leaves require less structural support per leaf area, indicating greater carbon allocation to photosynthesis, while the thinner boundary layer of small leaves permits more efficient heat and gas exchange and thus requires less cooling through transpirational water loss	Levionnois, Salmon et al. (2021), Michaletz et al. (2016), Wright et al. (2017), Ziegler et al. (2024)	Baraloto et al. (2010), Fortunel, Fine, and Baraloto (2012), Vleminckx et al. (2021)
Leaf	Specific leaf area	SLA (m^2^ kg^−1^)	Resource capture and defence	High SLA reflects greater allocation of dry mass to light interception than physical resistance and leaf lifespan and indicates acquisitive carbon use strategy	Osnas et al. (2013), Wright et al. (2004)	Baraloto et al. (2010), Fortunel, Fine, and Baraloto (2012), Vleminckx et al. (2021)
Leaf	Leaf thickness	*L*_thick_ (mm)	Resource capture and defence	High *L*_thick_ reflects greater allocation of dry mass to structural support, physical resistance and leaf lifespan and indicates conservative carbon use strategy	Vile et al. (2005)	Baraloto et al. (2010), Fortunel, Fine, and Baraloto (2012), Vleminckx et al. (2021)
Leaf	Leaf toughness	*L*_tough_ (N)	Resource capture and defence	High *L*_tough_ reflects greater allocation of dry mass to structural support, physical resistance and leaf lifespan and indicates conservative carbon use strategy	Kitajima and Poorter (2010)	Baraloto et al. (2010), Fortunel, Fine, and Baraloto (2012), Vleminckx et al. (2021)
Wood	Stem wood specific gravity	WSG	Stem transport, structure and defence	High wood specific gravity reflects greater allocation of dry mass to mechanical strength and resistance to abiotic and biotic threats and indicates conservative carbon use strategy and slow growth	Chave et al. (2009), Poorter et al. (2010)	Baraloto et al. (2010), Fortunel, Fine, and Baraloto (2012), Vleminckx et al. (2021)

## 2 Materials and Methods

### 2.1 Study Site and Inventory Data

This study leverages 30 years of spatially explicit inventory data from the CIRAD permanent forest plots of the Paracou research station (5°18′ N, 52°53′ W) in French Guiana. Paracou is a tropical lowland forest site with an annual precipitation of 3102 mm year^−1^ and a pronounced 3‐month dry season (< 100 mm month^−1^) spanning from mid‐August to mid‐November, during which wood production is reduced, and at the end of which water becomes limiting. Additionally, a shorter dry season can be observed in March (Aguilos et al. 2019).

The plot network was established between 1984 and 1990 and consists of fifteen 6.25 ha forest plots, covering 93.75 ha of predominantly terra‐firme forest. In 1987, nine plots were subjected to three intensities of silvicultural treatments including thinning, poison‐girdling and selective logging. These treatments resulted in 12%–56% loss of aboveground biomass (Gourlet‐Fleury, Guehl, and Laroussinie 2004), and led to contrasting community composition (Mirabel, Hérault, and Marcon 2020) and neighbourhood densities (Nemetschek et al. 2024) between plots and years. Since then, tree inventories took place every 2 years, during which the spatial location (precision 0.5 m), status (alive/dead) and circumference (precision 0.5 cm, from which we calculated diameter at breast height [DBH]), of each tree ≥ 10 cm DBH (i.e., 1.3 m) was recorded (Derroire, Hérault, et al. 2022; Gourlet‐Fleury, Guehl, and Laroussinie 2004). More than 590 species and subspecies, from 227 genera and 63 families have been measured at the site (mean 142 species per hectare), with the dominant families being Fabaceae, Chrysobalanaceae, Lecythidaceae, Sapotaceae and Burseraceae (Hérault et al. 2011).

We calculated individual annualised absolute diameter growth rate (AGR, cm year^−1^) from DBH at the end *t* and the start *t* − 2 of 15 two‐year census intervals between 1991 and 2021, excluding aberrant and uncertain growth measurements (see Methods S1 for details).

(1) $${\mathrm{AGR}}_{i,s,t}=\frac{{\mathrm{DBH}}_{i,s,t}-{\mathrm{DBH}}_{i,s,t-2}}{2}$$

Although most trees at Paracou were botanically identified, some individuals (< 10%) only received a vernacular name, mainly due to tree death before botanical identification could take place. To infer the most likely association between the botanical and vernacular name for a given individual, we used the vernabota R package (Derroire, Aubry‐Kientz, et al. 2022, see Methods S2 for details). While tree individuals with gapfilled species information were removed from the focal tree data, they were kept in the neighbourhood data (see Section 2.4).

### 2.2 Climate Data

To study the separate and interactive effects of climate and neighbours, we extracted mean monthly averages of three climate variables from the global high‐resolution TerraClimate data set (Abatzoglou et al. 2018): maximum temperature (*T*_max_), vapour pressure deficit (VPD) and climatic water deficit (CWD), which have been shown to capture tropical tree responses to different aspects of climate stress (Bauman, Fortunel, Cernusak, et al. 2022; Nemetschek et al. 2024). Specifically, these climate indices respectively capture heat stress, atmospheric evaporative demands and soil water availability, the latter by relating precipitation to evapotranspiration. We expressed interannual variation in these indices as the mean of monthly climate anomalies over each of the 2‐year census intervals, as follows (CA_*t*_, Figure S1): For each climate index and month, we calculated their deviations from their respective 30‐year monthly mean for the 1991–2021 period, before dividing them by their 30‐year monthly standard deviation (SD). We then averaged these standardised monthly climate anomalies over the 24 months prior to each census *t* (Bauman, Fortunel, Cernusak, et al. 2022; Nemetschek et al. 2024; Rifai et al. 2018, see Methods S3). The highest positive anomalies in *T*_max_ and VPD are more than a unit of SD (2019–2021), and about 0.5 SD for the highest anomaly in CWD (1997–1999) (Figures S1 and S2). Doing so allowed us to directly interpret climate induced growth variations as responses to higher climate stress than usual.

### 2.3 Trait Data

To capture species water relations (Table 1), we measured leaf water potential at turgor loss point (*π*_tlp_), leaf minimum conductance (*g*_min_) and leaf saturated water content (LSWC) in the dry seasons of 2020 and 2021 (Nemetschek et al. 2024). We selected target species according to their abundance to maximise neighbourhood coverage for our growth models. In addition, we combined our three water‐related traits with data from previous field campaigns at Paracou (Levionnois, Ziegler, et al. 2021; Maréchaux et al. 2015, 2019; Ziegler et al. 2019). We further compiled data on bulk leaf carbon isotope composition (δ^13^C), leaf area (LA), specific leaf area (SLA), leaf thickness (*L*_thick_), leaf toughness (*L*_tough_) and wood specific gravity (WSG) from previous work conducted in French Guiana (Baraloto et al. 2010; Fortunel, Fine, and Baraloto 2012; Vleminckx et al. 2021). We subsequently calculated species mean trait values from individual trait measurements. Our final trait dataset includes complete trait information on 89 species (from 71 genera and 34 families), that together represent 77% of all unique individual stems and 78% of growth measurements at Paracou. For more information on the different traits and data sources, see Table 1 and Nemetschek et al. (2024).

### 2.4 Neighbourhood Indices

For each individual focal tree *i* at the start of the growth census interval *t* − 2, we calculated three neighbourhood indices within a radius of 10 m around the focal tree (Fortunel et al. 2018; Lasky et al. 2014). To capture neighbourhood densities, we calculated a neutral neighbourhood crowding index (NCI) as:

(2) $${\mathrm{NCI}}_{i,t-2}=\sum_{j\in J(i)}\frac{{\mathrm{DBH}}_{j,t-2}^{2}}{d_{i,j}}$$

where *J*(*i*) are the neighbours of *i* (such that *i* ∉ *J*(*i*)) within the 10‐m radius and the influence of a given neighbour *j* on the focal tree *i* is proportional to its basal area $\left({\mathrm{DBH}}_{j}^{2}\right)$ and declines linearly with its distance (*d*_*ij*_) from the focal tree *i*.

To respectively capture the effects of trait hierarchies and dissimilarities between the focal tree and its neighbours, we calculated NIh and NId as the weighted average of trait hierarchies and dissimilarities between the focal tree and all its neighbours within the neighbourhood radius as:

(3) $$\begin{array}{c} {\mathrm{NIh}}_{i,t-2}=\frac{1}{{\mathrm{NCI}}_{i,t-2}}\times \left(\sum_{k=1}^{K}\lambda_{s,k}\sum_{j\in J_{k}(i)}\frac{{\mathrm{DBH}}_{j,t-2}^{2}}{d_{i,j}}\right) \end{array}$$

(4) $$\begin{array}{c} {\mathrm{NId}}_{i,t-2}=\frac{1}{{\mathrm{NCI}}_{i,t-2}}\times \left(\sum_{k=1}^{K}|\lambda_{s,k}|\sum_{j\in J_{k}(i)}\frac{{\mathrm{DBH}}_{j,t-2}^{2}}{d_{i,j}}\right) \end{array}$$

where trait hierarchies are relative trait differences $\left(\lambda_{s,k}={\text{trait}}_{s}-{\text{trait}}_{k}\right)$ and trait dissimilarities are absolute trait differences $\left(|,\lambda_{s,k},|,=,\left|{\text{trait}}_{s}-{\text{trait}}_{k}\right|\right)$ between the species *s* of focal tree *i* and the species *k* of its *J*_*k*_(*i*) ⊆ *J*(*i*) neighbours *j*. *λ*_*s,k*_ increasingly differs from 0 with increasing relative (hierarchical) and absolute (dissimilarities) trait differences (Lasky et al. 2014). The contribution of trait differences (*λ*_*s,k*_) between the focal tree and each neighbour *j* to NIh and NId is weighted by the squared diameter of *j* and its inverse distance *d*_*ij*_ to the focal tree *i* (i.e., its contribution to the NCI). For a given focal tree, NIh therefore increases when the focal tree has a relatively higher trait value in comparison with its neighbour and decreases when the focal tree has a relatively lower trait value in comparison with its neighbour. NId increases with increasing absolute trait differences (dissimilarities) between the focal and its neighbours. These weighted averages of trait differences, NIh and NId, are therefore not influenced by the density of neighbours, which is further shown by their very low correlation with NCI (see Table S1).

The 89 species for which complete information for all nine traits was available constitute our focal species. As NIh and NId require trait information for all neighbours within the neighbourhood, we gapfilled missing trait information for all remaining species using the year and plot specific community weighted mean. To reduce the influence of missing species trait information on neighbourhood effect estimates, we only selected focal trees for which at least 75% of their NCI belonged to species with available trait information. For more detailed information on neighbourhood indices and subsetting of focal individuals, see Methods S4.

### 2.5 Models

We evaluated the separate and interactive effects of climate anomalies and neighbourhood indices (NCI, NIh and NId) on individual absolute growth rates (AGR) using hierarchical Bayesian models. To manage model complexity, we fitted models separately for each combination of (i) trait hierarchies (NIh) and dissimilarities (NId), (ii) the three climate variables (*T*_max_, VPD and CWD) and (iii) the nine functional traits, resulting in a total of 54 models. The model hierarchy consists of a community‐level regression and a species‐level response. The community‐level regression models AGR responses to covariates via hyperparameters (i.e., statistical distributions from which species‐level intercepts and slope coefficients arose), whereas the species‐level captures species deviations from the community average parameters.

For each individual *i* of species *s* in plot *p* between censuses *t* − 2 and *t*, we modelled the logarithm of scaled and centred absolute tree growth with a normal distribution as:

(5a) $$\log\left({\mathrm{AGR}}_{i,s,t,p}\right)∼N\left(\mu_{i,s,t,p},\sigma^{2}\right)$$

where the mean $\mu_{i,s,t,p}$ is a linear function of the natural logarithm of scaled and centred tree size within species at the beginning of the census interval (DBH_*i*,*t* − 2_), monthly climate anomalies averaged over the census interval (CA_*t*_), the natural logarithm of scaled and centred neutral neighbourhood crowding index (NCI_*i*,*t* − 2_), one of the non‐neutral neighbourhood index (NI_*i*,*t* − 2_) capturing either trait hierarchies (NIh_*i*,*t* − 2_) or trait dissimilarities (NId_*i*,*t* − 2_) at the beginning of the census interval and their interactive effects with climate anomalies (CA_*t*_ × NCI_*i*,*t* − 2_ and CA_*t*_ × NI_*i*,*t* − 2_) (see details on variable transformation in Methods S5) as:
(5b)

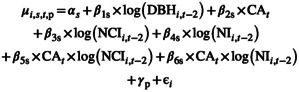


*α*_s_ and *β*_1–6s_ are species‐specific coefficients representing intrinsic AGR (*α*_s_), and species responses to tree size (*β*_1s_), climate anomalies (*β*_2s_), neighbourhood crowding (*β*_3s_), hierarchical or dissimilarity neighbourhood index (*β*_4s_) as well as interactive effect of climate anomalies with neighbourhood crowding index (*β*_5s_), or with hierarchical or dissimilarity neighbourhood index (*β*_6s_). We further allowed intercepts to vary by plots $\gamma_{p}$ and individuals *ϵ*_*i*_, to capture part of the unexplained growth variation related to plots and individuals (Bauman, Fortunel, Cernusak, et al. 2022; Fortunel et al. 2018).

Species intrinsic AGR *α*_s_ and AGR response to covariates *β*_1–6s_ for the *s* species were modelled using a multivariate normal distribution:

(5c) $$\left(\begin{array}{c} \alpha_{s}\\ \beta_{1s}\\ \vdots \\ \beta_{6s} \end{array}\right)∼\mathrm{MV}\;\text{Normal}\left[\left(\begin{array}{c} \alpha \\ \beta_{1}\\ \vdots \\ \beta_{6} \end{array}\right),S\right]$$

where *α* represents the community‐level intrinsic growth rate, *β*_1–6_ the overall effect of covariates on AGR across all species and *S* is a covariance matrix. Modelling all species‐level parameters as a multivariate normal distribution allows sharing information across species, thus improving the fit for poorly represented species, while preventing overfitting (McElreath 2020). For the full model equation and the specified weakly informative priors see Methods S6.

Models were fitted in the R environment (R Core Team 2021; RStudio Team 2020) on the Meso@LR HPC cluster using the package brms (Bürkner 2017). Bayesian updating of parameters was performed via the No‐U‐Turn Sampler (NUTS) in Stan (Carpenter et al. 2017) using CmdStanR (Stan Development Team 2022). We used four chains and 3000 iterations (1500 warm up) per chain. Chains of all models mixed well and generally converged within 1500 iterations (Rhat between 1 and 1.05). Model parameter posteriors were summarised through their median and 95% highest posterior density interval (HPDI) using the packages tidyverse (Wickham et al. 2019) and tidybayes (Kay 2022). Model covariates were considered to have a clear effect when the slope coefficients 95% HPDIs did not encompass zero. Additionally, effects of model covariates are discussed as trends when the slope coefficients 90% HPDIs did not encompass zero. For details on parameter estimates see Table S2. To assess the model goodness of fit, we calculated a Bayesian version of conditional and marginal *R* ^2^ (Gelman et al. 2019), which represent respectively the fraction of variance explained by the fixed and random terms and by the fixed terms only, using the bayes_R2() function of the brms package (Bürkner 2017). Our models had a high explanatory power, with a mean conditional *R* ^2^ of 61% (Table S3) and showed to be stable across climate‐trait model combinations (Methods S7). Some species showed clear deviations from the community‐level response to neighbourhood trait differences (NId and NIh), but not to their interaction with climate (Table S4; Figure S6). To ensure that observed effects to NId and NIh were not simply driven by focal tree trait values, we calculated pairwise Pearson correlations between species‐specific focal tree trait values and their responses to the separate and interactive effects of neighbourhood trait differences and climate anomalies (Table S5). The few significant correlations we found were weak, suggesting that observed effects are largely attributable to trait differences between neighbours, and species‐specific deviations from the community mean are driven by factors other than the traits considered in this study. Furthermore, we ensured that effects of neighbourhood trait dissimilarities were not merely a result of higher densities of conspecific neighbours, by showing the absence of strong correlations between NId and the contribution of conspecific neighbours to NCI (Figure S3). Lastly, we checked that our results were not driven by spatial autocorrelation by showing the absence of strong correlations among residuals of all 54 models (for details see Methods S8; Table S6).

## 3 Results

### 3.1 Tree Growth Response to Neighbourhood Indices

**FIGURE 2:**
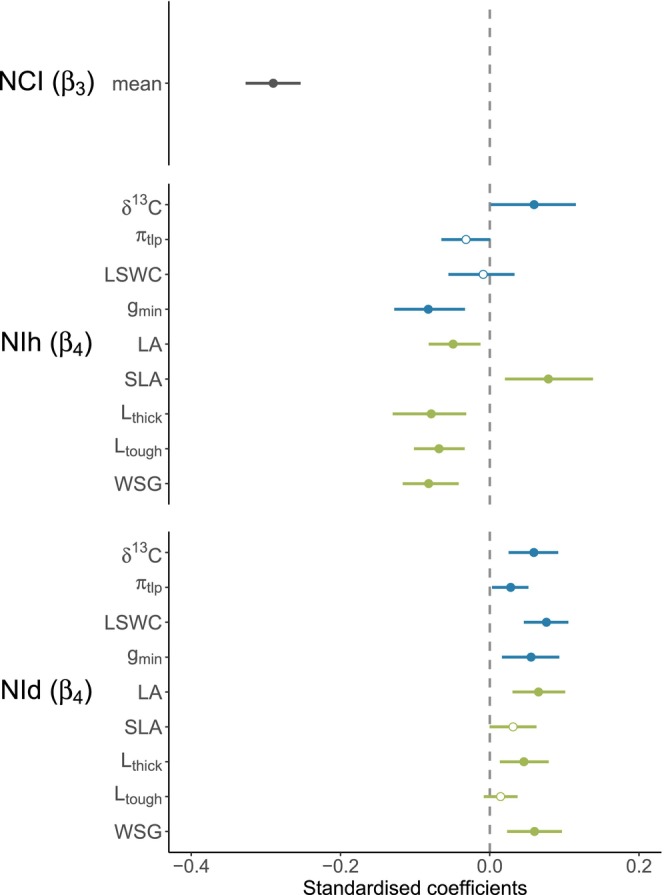
Community‐level effect of neutral neighbourhood crowding (NCI, *β*_3_), hierarchical (NIh, *β*_4_) and dissimilarity (NId, *β*_4_) neighbourhood index on tree growth. Standardised coefficients where highly stable across the three climate models (*T*_max_, VPD and CWD) and are therefore here only shown for the *T*_max_ models. Standardised coefficients are shown for NCI as mean estimates across the two NI and nine trait models and for NIh and NId separately for each of the nine trait models: Carbon (δ^13^C) isotope composition, water potential at turgor loss point (*π*_tlp_), leaf saturated water content (LSWC), minimum conductance (*g*_min_), leaf area (LA), specific leaf area (SLA), leaf thickness (*L*_thick_), leaf toughness (*L*_tough_) and wood specific gravity (WSG). For separate estimates for each model see Figures S4 and S5. Blue and green colours denote traits pertaining to water relations and carbon use, respectively. Circles show posterior medians of standardised coefficients, and lines indicate 95% HPDIs. Model covariates were considered to have a clear effect when the slope coefficients 95% HPDIs did not encompass zero. Filled circles indicate clear negative and positive effects (i.e., slope coefficient 95% HPDI not encompassing zero), and empty circles indicate no clear effects. Positive *β*_3–4_ values indicate faster growth with increasing neighbourhood index, while negative *β*_3–4_ values indicate slower growth with increasing neighbourhood index (details in Table S2).

Individual tree growth strongly declined (negative *β*_3_) with increasing NCI (neutral neighbourhood crowding), while effect sizes were smaller for both NIh (trait hierarchies) and NId (trait dissimilarities, Figure 2). Depending on the trait greater NIh either increased (positive *β*_4_) or reduced (negative *β*_4_) tree growth, while greater NId consistently increased (positive *β*_4_) it. More specifically, higher NIh in δ^13^C and SLA increased growth, indicating that focal trees grew faster when their intrinsic water use efficiency and specific leaf area was higher than those of their neighbours. On the other hand, growth declined with increasing NIh in *π*_tlp_, *g*_min_, LA, *L*_thick_, *L*_tough_ or WSG, indicating that focal trees grew slower when they had higher water potential at turgor loss point, higher minimum conductance, larger, thicker or tougher leaves as well as higher wood specific gravity than that of their neighbours. Lastly, higher NId in δ^13^C, *π*_tlp_, LSWC, *g*_min_, LA, SLA (trend observed for 90% HDPI but not for 95%), *L*_thick_ and WSG positively influenced tree growth, indicating trees grew faster when their neighbours were more dissimilar in these trait values.

### 3.2 Tree Growth Response to Interactive Effects of Climate Anomalies and Neighbourhood Indices

**FIGURE 3:**
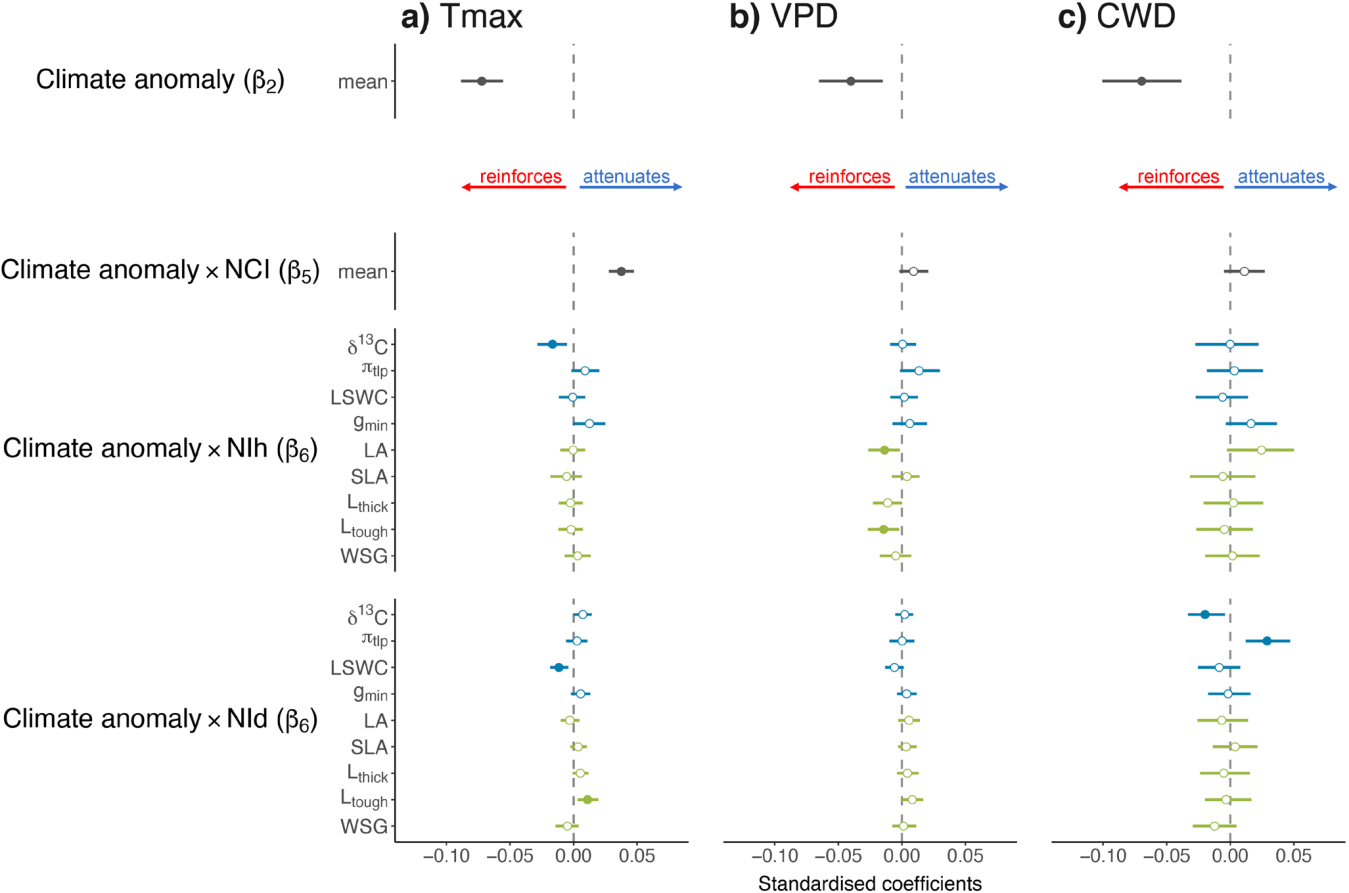
Community‐level effects of climate anomaly (*β*_2_), and the interactions between climate anomaly and neutral neighbourhood crowding (Climate anomaly NCI, *β*_5_), hierarchical (Climate anomaly NIh, *β*_6_) and dissimilarity (Climate anomaly NId, *β*_6_) neighbourhood index on tree growth. Standardised coefficients from (a) *T*_max_, (b) VPD and (c) CWD models are shown for climate anomalies and its interaction with NCI as mean estimates across the two NI and nine trait models (see Figures S4 and S5 for separate estimates) and for the interaction between climate anomaly and NIh and NId separately for each of the nine trait models: Carbon (δ^13^C) isotope composition, water potential at turgor loss point (*π*_tlp_), leaf saturated water content (LSWC), minimum conductance (*g*_min_), leaf area (LA), specific leaf area (SLA), leaf thickness (*L*_thick_), leaf toughness (*L*_tough_) and wood specific gravity (WSG). Blue and green colours denote traits pertaining to water relations and carbon use, respectively. Circles show posterior medians of standardised coefficients, and lines indicate 95% HPDIs. Model covariates were considered to have a clear effect when the slope coefficients 95% HPDIs did not encompass zero. Filled circles indicate clear negative and positive effects (i.e., slope coefficient 95% HPDI not encompassing zero), and empty circles indicate no clear effects. Positive *β*_5–6_ values indicate a buffering effect of either NCI or NI, while negative *β*_5–6_ values indicate an accentuating effect of either NCI or NI (details in Table S2).

Positive anomalies in *T*_max_, VPD and CWD reduced tree growth (negative *β*_2_). Moreover, higher NCI led to a clear buffering (positive *β*_5_, Figure 3) of negative effects of *T*_max_, while also showing a strong trend to buffer negative effects of VPD and CWD. Only a few trait differences between the focal tree and its neighbours led to a clear modulation of growth through trait hierarchies (NIh) or dissimilarities (NId), and these effects depended on the climate variable. More specifically, negative effects of *T*_max_ were reinforced (negative *β*_6_) for trees with relatively higher δ^13^C than their neighbours (higher NIh). On the other hand, negative effects of *T*_max_ tended to be buffered (positive *β*_6_) for trees with relatively higher *π*_tlp_ (trend) and *g*_min_ (trend) than their neighbourhood (higher NIh). Furthermore, negative effects of *T*_max_ were reinforced (negative *β*_6_) with increasing trait dissimilarities (higher NId) in LSWC but tended to be attenuated (positive *β*_6_) for trees surrounded by more dissimilar neighbours regarding δ^13^C (trend), *L*_thick_ (trend) and *L*_tough_ (Figure 3a).

While increasing trait hierarchies in LA, *L*_thick_ (trend) and *L*_tough_ reinforced the negative effects of VPD on tree growth (negative *β*_6_), increasing trait hierarchies in *π*_tlp_ (trend) and greater dissimilarity (higher NId) in *L*_tough_ (trend) tended to buffer them (positive *β*_6_, Figure 3b).

Lastly, while focal trees with more dissimilar neighbourhoods regarding *π*_tlp_ (higher NId) were less affected by higher CWD, greater dissimilarities in δ^13^C (higher NId) accentuated negative effects of CWD (negative *β*_6_). Additionally, focal trees tended to be less affected (positive *β*_6_) from higher CWD when having relatively higher LA (trend) than their neighbours (higher NIh, Figure 3c).

## 4 Discussion

By combining 30 years of high temporal resolution census data with data on neighbourhood trait differences pertaining to plant–water relations and carbon use (Table 1) from a highly diverse tropical forest, our study indicates that neutral and asymmetric competition likely act in concert with resource partitioning to shape tropical tree growth (Figure 1) and its response to heat (*T*_max_), atmospheric (VPD) and soil water drought (CWD) stress (Figure 2). Building on our previous study showing that focal tree traits can explain species' sensitivity to neighbourhood densities, climate and to some extend their interactive effects (Nemetschek et al. 2024), we here provide novel insights on the potential mechanisms underlying positive and negative neighbourhood interactions in both normal and anomalous climate years, by focusing on neighbourhood trait difference mediated effects.

Neighbourhood crowding (NCI), capturing neighbourhood density, strongly reduced individual tree growth (Figure 1) and had by far the largest effect size of any of the six tested model covariates. This suggests that competition for shared space and resources is a key driver of tree growth at Paracou (Nemetschek et al. 2024) and that competitive interactions between neighbouring trees are foremost driven by their size and proximity in space (Laurans et al. 2014; Moravie, Pascal, and Auger 1997). Previous studies have shown that neighbourhood taxonomic diversity can positively influence tree performance, likely through increased functional dissimilarity between neighbours promoting resource partitioning (Ammer 2019; Forrester and Bauhus 2016). Our results provide direct and strong support of this hypothesis as greater dissimilarity in all nine tested traits consistently stimulated individual tree growth at our site (Figure 2). Greater dissimilarity between neighbours in traits related to carbon use and root strategies have also previously been shown to increase tropical and subtropical tree performance (Fortunel et al. 2016; Huang et al. 2022; Lasky et al. 2014; Uriarte et al. 2010). Here we show for the first time that this extends to traits pertaining to leaf water relations (δ^13^C, *π*_tlp_, LSWC, *g*_min_), uncovering the importance of complementary water use and drought response strategies even in predominantly light‐ rather than water‐limited tropical forests such as Paracou (Wagner et al. 2016). This complementarity may be especially beneficial when a shift from light to water limitation can be observed (Meng et al. 2022), such as during the pronounced dry season at Paracou (Figure S2).

Providing further evidence for the importance of interactions for water at the neighbourhood scale, we show that greater trait hierarchies in water‐related traits, here used to capture asymmetric neighbourhood interactions (Figure 1), significantly influence tree growth (Figure 2). Our results suggest that a higher water use efficiency (higher δ^13^C), a greater ability to maintain physiological functioning under decreasing water availability (more negative *π*_tlp_) and conserve water under drought stress (lower *g*_min_) than its neighbours may provide a competitive advantage, as reflected by faster growth. Contrasting to our findings for water‐related traits and previous research (Fortunel et al. 2016; Kunstler et al. 2016), greater conservatism in carbon use relative to neighbours (lower SLA and greater *L*_thick_, *L*_tough_ and WSG) was consistently associated with reduced tree growth. This highlights that greater resource conservatism in comparison with neighbours does not always result in a competitive advantage in tropical forests. Having more conservative carbon use strategies than one's neighbours implies being surrounded by more resource‐acquisitive neighbours that may faster deplete common resources (Garbowski et al. 2020; Goldberg 1990). Specifically, faster growth at the expense of less mechanically resistant leaf and wood tissue (Chave et al. 2009; Reich 2014) promotes fast colonisation of forest gaps both vertically and horizontally (Westoby et al. 2002), which constitutes a strong competitive advantage in disturbed plots making up 51% of growth observations at Paracou.

In line with our findings in Nemetschek et al. (2024), denser neighbourhoods consistently attenuated negative climate effects on tree growth (Figure 3) likely through sheltering trees from atmospheric climate extremes, thereby improving local microclimatic conditions (De Frenne et al. 2019; Tymen et al. 2017; Wright 2024). Simultaneously, neighbourhood taxonomic diversity can influence growth responses to drought (Grossiord 2020). If resource partitioning is a key driver of positive diversity effects on drought resistance, their magnitude should depend on the functional identity of focal trees (Fichtner et al. 2020), that of their neighbours (Schnabel et al. 2024) and ultimately on their functional differences. Our results suggest that in certain cases greater trait dissimilarities can indeed increase individual growth resistance to climate stress. However, this effect can differ largely across traits and climate variables. Greater dissimilarities in leaf economics traits tended to buffer negative effects of the atmospheric climate variables *T*_max_ (for *L*_thick_ and *L*_tough_ [trend]) and to a lesser extend for VPD (for *L*_thick_ [trend]). Increased complementarity in leaf morphology could indicate greater canopy space filling (Forrester and Bauhus 2016, but see Hildebrand et al. 2021), which likely increases thermal insulation (De Frenne et al. 2019; Zhang et al. 2022). Conversely, greater dissimilarities in *π*_tlp_ mitigated negative effects of soil water stress (CWD). *π*_tlp_ is a key drought tolerance trait (Bartlett, Scoffoni, and Sack 2012) and a strong predictor of leaf water potential at stomatal closure (Martin‐StPaul, Delzon, and Cochard 2017; Rodriguez‐Dominguez et al. 2016). This finding aligns with previous studies showing that diversity in stomatal regulation and drought response strategies can reduce plant–water stress by positively affecting local soil moisture (Grossiord 2020; Moreno et al. 2023). However, in contrast to our expectations, we observed that greater trait dissimilarities in water use efficiency (as indicated by 13C) reinforced negative effects of soil water stress (CWD). While *π*_tlp_ and δ^13^C can inform on species' water use strategies, the two traits (Maréchaux et al. 2019) and thus resulting neighbourhood indices (Figure S3) were found largely uncorrelated in French Guyanese forests. These contrasting results, therefore highlight their different functions (Table 1) in influencing tree water‐status and neighbourhood interactions. Additionally, intertree variation in water use and thus neighbourhood interactions for water may also depend on rooting depth, which has been shown to vary independently of tree size in Paracou (Stahl et al. 2013) and may influence the relationship between leaf‐ and whole‐tree level water use.

Even in normal climate years, trees at Paracou still experience great exposure to heat, high atmospheric water demands and low soil water availability, reaching its peak during the dry season (Figure 2). Interestingly, our results indicate that the competitive advantage of water‐saver species observed in normal years decreases in extreme climate years, as the negative effect of a higher water consumption by the neighbourhood becomes more important (Figures 2 and 3). Specifically, greater wateruse efficiency (higher δ^13^C) relative to neighbours benefited individual tree growth in normal years, but reinforced negative effects of temperature stress (*T*_max_). Higher temperatures can lead to increased evapotranspiration, hence greater abundances of relatively less water‐saver neighbours likely exert greater pressure on local soil water resources when temperature stress occurs (Grossiord et al. 2014; Mas et al. 2024). Conversely, trees with larger LA relative to their neighbours grew slower in normal years but suffered less from negative effects of soil water (CWD) stress. While larger leaves require less structural support per leaf area, indicating greater carbon allocation to photosynthesis (Michaletz et al. 2016), they require more cooling through (higher) transpirational water loss, which necessitates greater water supply per unit leaf area (Wright et al. 2017). Similarly, trees with higher residual water loss (*g*_min_) also tended to be more buffered against negative effects of temperature stress (*T*_max_). Our results therefore suggest a positive effect of water‐saver species on local soil water availability during drought and heat waves, which may particularly benefit water–spender species (Mas et al. 2024; Moreno et al. 2023). These findings can also provide mechanistic insights to why species with low drought tolerance profit most from neighbourhood diversity during drought, as shown by Fichtner et al. (2020). Conversely our results indicate that water–spender tree neighbours likely decrease water resources to the detriment of the focal tree (Garbowski et al. 2020; Goldberg 1990), increasing the climate stress experienced particularly for water‐saver species.

As forest ecosystems are increasingly likely to experience environmental conditions beyond their normal range, understanding if currently observed biotic interactions will hold in a changing climate is crucial (Grossiord et al. 2019). By considering neighbourhood differences in water‐related traits in addition to carbon‐related ones, our study shows that the consistent positive effect of resource partitioning, here inferred from neighbourhood dissimilarities, observed under normal conditions becomes more complex in climatically stressful years. Similarly, our results suggest that trees profit from greater water‐saving capacities relative to their neighbours in normal years but as climate stress increases, become increasingly affected by their neighbours' overall water consumption. Our results, in combination with the findings of our previous study (Nemetschek et al. 2024), highlighting the importance of focal tree traits in explaining tropical tree growth and response to climate, strongly suggest that climate change adapted forest management should carefully consider species' differences in water use strategies in combination with neighbourhood interactions (Forrester et al. 2016). While we previously showed that focal tree traits can explain species sensitivities to neighbourhood densities (Nemetschek et al. 2024), in the present study species‐specific responses to neighbourhood trait differences and their interaction with climate were not merely driven by the here considered trait values of the focal tree (see Table S5). Furthermore, except for certain carbon use traits, traits mediating focal tree growth and response to climate in Nemetschek et al. (2024) did not have the same effect when their neighbourhood differences were considered in the present study (see Table S7). This stresses the importance of also considering the functional identity of the focal tree in relation to its neighbours to better understand patterns of tree growth and climate response in diverse tropical forests. Having said this, across trait models, some species showed clear and consistent deviations from the community‐level response to NId and NIh, but not their interaction with climate (Table S4; Figure S6). Preliminary exploration of these interspecific differences in responses revealed that the strength and direction of species responses to neighbourhood trait differences may be driven by species' succession status, as early successional species tended to deviated more strongly from the community‐level response (Figure S6). We therefore stress the importance of moving beyond the taxonomic diversity lens to understand how different types of neighbourhood interactions affect tree performance in these new conditions, which provides a promising way forward to assess the productivity and resilience of entire forest ecosystems under climate change.

## Author Contributions

D.N., G.D., C.F. and E.M. designed the study. D.N., M.B., J.A., V.B., C.B., D.B., S.C., E.D., C.F., P.H., P.H., S.L., I.M., C.S., J.V., W.W. and C.Z. collected or contributed trait data. D.N. formatted and vetted the plot census, climate and functional trait data, with help from G.D., C.F., E.M. and M.B. D.N., G.D., C.F. and E.M. designed the tree growth models. D.N. performed the analyses, with help from G.D., C.F. and E.M. D.N. and G.D. led the interpretation of the results, and D.N. wrote the first draft, with frequent discussions with G.D. and inputs from C.F. and E.M. All authors contributed substantially to revisions and gave final approval for publication.

### Peer Review

The peer review history for this article is available at https://www.webofscience.com/api/gateway/wos/peer‐review/10.1111/ele.70028.

## Supporting information


**Figure S1.** Adapted figure from Nemetschek et al. (2024): Mean standardised climate anomalies at Paracou for the two‐year census intervals over the study period.
**Figure S2.** Absolute values of mean monthly climate indices in comparison with their respective 30‐year monthly mean for the 1991–2021 period.
**Figure S3.** Correlation matrix showing pairwise Pearson correlation coefficients between all neighbourhood indices.
**Figure S4.** Standardised regression coefficients of community‐level parameter estimates from NIh models.
**Figure S5.** Standardised regression coefficients of community‐level parameter estimates from NId models.
**Figure S6.** Overview of species‐level responses to NIh and NId that clearly deviate from the community‐level response.
**Methods S1.** Corrections of tree inventory data.
**Methods S2.** Gapfilling of missing species information.
**Methods S3.** Calculation of climate anomalies.
**Methods S4.** Additional information on neighbourhood indices.
**Methods S5.** Transformation of response variable and model covariates.
**Methods S6.** Full model equation.
**Methods S7.** Information on model stability.
**Methods S8.** Spatial autocorrelation check.

**Table S1.** Pairwise Pearson correlation coefficients between weighted and unweighted neighbourhood indices.
**Table S2.** Standardised regression coefficients of community‐level parameters and group‐level sigmas.
**Table S3.** Conditional and marginal *R* ^2^ estimates models.
**Table S4.** Summary table for species‐level responses to non‐neutral neighbourhood indices (NIh and NId) and their interaction with climate anomalies.
**Table S5.** Pairwise Pearson correlation coefficients between species trait values and species‐level responses to non‐neutral neighbourhood indices (NIh and NId) and their interaction with climate anomalies.
**Table S6.** Summary table for spatial autocorrelation check.
**Table S7.** Comparison table of trait‐mediated effects between Nemetschek et al. (2024), Journal of Ecology and the present study.

## Data Availability

Data and R Code to reproduce results, figures and tables represented in this manuscript are available at https://doi.org/10.5281/zenodo.10719861.

## References

[ele70028-bib-0001] Abatzoglou, J. T. , S. Z. Dobrowski , S. A. Parks , and K. C. Hegewisch . 2018. “TerraClimate, a High‐Resolution Global Dataset of Monthly Climate and Climatic Water Balance From 1958–2015.” Scientific Data 5: 170191. 29313841 10.1038/sdata.2017.191 PMC5759372

[ele70028-bib-0002] Aguilos, M. , C. Stahl , B. Burban , et al. 2019. “Interannual and Seasonal Variations in Ecosystem Transpiration and Water Use Efficiency in a Tropical Rainforest.” Forests 10: 14.

[ele70028-bib-0003] Allen, C. D. , A. K. Macalady , H. Chenchouni , et al. 2010. “A Global Overview of Drought and Heat‐Induced Tree Mortality Reveals Emerging Climate Change Risks for Forests.” Forest Ecology and Management 259: 660–684.

[ele70028-bib-0004] Ammer, C. 2019. “Diversity and Forest Productivity in a Changing Climate.” New Phytologist 221: 50–66. 29905960 10.1111/nph.15263

[ele70028-bib-0005] Anderegg, W. R. L. , A. G. Konings , A. T. Trugman , et al. 2018. “Hydraulic Diversity of Forests Regulates Ecosystem Resilience During Drought.” Nature 561: 538–541. 30232452 10.1038/s41586-018-0539-7

[ele70028-bib-0006] Aschehoug, E. T. , R. Brooker , D. Z. Atwater , J. L. Maron , and R. M. Callaway . 2018. “The Mechanisms and Consequences of Interspecific Competition.” Annual Review of Ecology and Systematics 47: 263–281.

[ele70028-bib-0007] Baraloto, C. , C. E. Timothy Paine , L. Poorter , et al. 2010. “Decoupled Leaf and Stem Economics in Rain Forest Trees.” Ecology Letters 13: 1338–1347. 20807232 10.1111/j.1461-0248.2010.01517.x

[ele70028-bib-0008] Bartlett, M. K. , C. Scoffoni , and L. Sack . 2012. “The Determinants of Leaf Turgor Loss Point and Prediction of Drought Tolerance of Species and Biomes: A Global Meta‐Analysis.” Ecology Letters 15: 393–405. 22435987 10.1111/j.1461-0248.2012.01751.x

[ele70028-bib-0009] Bauman, D. , C. Fortunel , L. A. Cernusak , et al. 2022. “Tropical Tree Growth Sensitivity to Climate Is Driven by Species Intrinsic Growth Rate and Leaf Traits.” Global Change Biology 28: 1414–1432. 34741793 10.1111/gcb.15982

[ele70028-bib-0010] Bauman, D. , C. Fortunel , G. Delhaye , et al. 2022. “Tropical Tree Mortality Has Increased With Rising Atmospheric Water Stress.” Nature 608: 528–533. 35585230 10.1038/s41586-022-04737-7

[ele70028-bib-0011] Begon, M. , C. R. Townsend , and J. L. Harper . 2006. Ecology: From Individuals to Ecosystems. 4th ed. Malden: Blackwell Publishing Ltd.

[ele70028-bib-0012] Belote, R. T. , S. Prisley , R. H. Jones , M. Fitzpatrick , and K. de Beurs . 2011. “Forest Productivity and Tree Diversity Relationships Depend on Ecological Context Within Mid‐Atlantic and Appalachian Forests (USA).” Forest Ecology and Management 261: 1315–1324.

[ele70028-bib-0116] Blackman, C. J. , D. Creek , C. Maier , et al. 2019. “Drought Response Strategies and Hydraulic Traits Contribute to Mechanistic Understanding of Plant Dry‐Down to Hydraulic Failure.” Tree Physiology 39, no. 6: 910–924. 30865274 10.1093/treephys/tpz016

[ele70028-bib-0013] Bottero, A. , A. W. D'Amato , B. J. Palik , et al. 2017. “Density‐Dependent Vulnerability of Forest Ecosystems to Drought.” Journal of Applied Ecology 54: 1605–1614.

[ele70028-bib-0014] Brodribb, T. J. 2017. “Progressing From ‘Functional’ to Mechanistic Traits.” New Phytologist 215: 9–11. 28560790 10.1111/nph.14620

[ele70028-bib-0015] Brooker, R. W. , F. T. Maestre , R. M. Callaway , et al. 2007. “Facilitation in Plant Communities: The Past, the Present, and the Future.” Journal of Ecology 96: 18–34.

[ele70028-bib-0016] Bürkner, P. C. 2017. “brms: An R Package for Bayesian Multilevel Models Using Stan.” Journal of Statistical Software 80: 1–28.

[ele70028-bib-0017] Callaway, R. M. 1995. “Positive Interactions Among Plants.” Botanical Review 61: 306–349.

[ele70028-bib-0018] Canham, C. D. , P. T. LePage , and K. D. Coates . 2004. “A Neighborhood Analysis of Canopy Tree Competition: Effects of Shading Versus Crowding.” Canadian Journal of Forest Research 34: 778–787.

[ele70028-bib-0019] Carpenter, B. , A. Gelman , M. D. Hoffman , et al. 2017. “Stan: A Probabilistic Programming Language.” Journal of Statistical Software 76: 1–32. 36568334 10.18637/jss.v076.i01 PMC9788645

[ele70028-bib-0020] Cernusak, L. A. , N. Ubierna , K. Winter , J. A. M. Holtum , J. D. Marshall , and G. D. Farquhar . 2013. “Environmental and Physiological Determinants of Carbon Isotope Discrimination in Terrestrial Plants.” New Phytologist 200: 950–965. 23902460 10.1111/nph.12423

[ele70028-bib-0021] Chave, J. , D. Coomes , S. Jansen , S. L. Lewis , N. G. Swenson , and A. E. Zanne . 2009. “Towards a Worldwide Wood Economics Spectrum.” Ecology Letters 12: 351–366. 19243406 10.1111/j.1461-0248.2009.01285.x

[ele70028-bib-0022] Craine, J. M. , and R. Dybzinski . 2013. “Mechanisms of Plant Competition for Nutrients, Water and Light.” Functional Ecology 27: 833–840.

[ele70028-bib-0023] Crawford, M. S. , K. E. Barry , A. T. Clark , et al. 2021. “The Function‐Dominance Correlation Drives the Direction and Strength of Biodiversity–Ecosystem Functioning Relationships.” Ecology Letters 24: 1762–1775. 34157796 10.1111/ele.13776

[ele70028-bib-0024] De Frenne, P. , F. Rodríguez‐Sánchez , D. A. Coomes , et al. 2013. “Microclimate Moderates Plant Responses to Macroclimate Warming.” Proceedings of the National Academy of Sciences of the United States of America 110: 18561–18565. 24167287 10.1073/pnas.1311190110 PMC3832027

[ele70028-bib-0025] De Frenne, P. , F. Zellweger , F. Rodríguez‐Sánchez , et al. 2019. “Global Buffering of Temperatures Under Forest Canopies.” Nature Ecology & Evolution 3: 744–749. 30936433 10.1038/s41559-019-0842-1

[ele70028-bib-0026] Delzon, S. 2015. “New Insight Into Leaf Drought Tolerance.” Functional Ecology 29: 1247–1249.

[ele70028-bib-0027] Derroire, G. , M. Aubry‐Kientz , A. Mirabel , E. Marcon , and H. Bruno . 2022. “vernabota: Association between vernacular and botanical names for Guyafor data.” R package. https://ecofog.github.io/vernabota/.

[ele70028-bib-0028] Derroire, G. , B. Hérault , V. Rossi , L. Blanc , S. Gourlet‐Fleury , and L. Schmitt . 2022. “Paracou Biodiversity Plots.”

[ele70028-bib-0029] Duursma, R. A. , C. J. Blackman , R. Lopéz , N. K. Martin‐StPaul , H. Cochard , and B. E. Medlyn . 2019. “On the Minimum Leaf Conductance: Its Role in Models of Plant Water Use, and Ecological and Environmental Controls.” New Phytologist 221: 693–705. 30144393 10.1111/nph.15395

[ele70028-bib-0030] Farquhar, G. D. , J. R. Ehleringer , and K. T. Hubick . 1989. “Carbon Isotope Discrimination and Photosynthesis.” Annual Review of Plant Physiology and Plant Molecular Biology 40: 503–537.

[ele70028-bib-0031] Fichtner, A. , W. Härdtle , H. Bruelheide , M. Kunz , Y. Li , and G. Von Oheimb . 2018. “Neighbourhood Interactions Drive Overyielding in Mixed‐Species Tree Communities.” Nature Communications 9: 1–8. 10.1038/s41467-018-03529-w PMC5861250 29559628

[ele70028-bib-0032] Fichtner, A. , F. Schnabel , H. Bruelheide , et al. 2020. “Neighbourhood Diversity Mitigates Drought Impacts on Tree Growth.” Journal of Ecology 108: 865–875.

[ele70028-bib-0033] Forrester, D. I. , and J. Bauhus . 2016. “A Review of Processes Behind Diversity—Productivity Relationships in Forests.” Current Forestry Reports 2: 45–61.

[ele70028-bib-0034] Forrester, D. I. , D. Bonal , S. Dawud , et al. 2016. “Drought Responses by Individual Tree Species Are Not Often Correlated With Tree Species Diversity in European Forests.” Journal of Applied Ecology 53: 1725–1734.

[ele70028-bib-0035] Fortunel, C. , P. V. A. Fine , and C. Baraloto . 2012. “Leaf, Stem and Root Tissue Strategies Across 758 Neotropical Tree Species.” Functional Ecology 26: 1153–1161.

[ele70028-bib-0036] Fortunel, C. , J. R. Lasky , M. Uriarte , et al. 2018. “Topography and Neighborhood Crowding Can Interact to Shape Species Growth and Distribution in a Diverse Amazonian Forest.” Ecology 99: 2272–2283. 29975420 10.1002/ecy.2441

[ele70028-bib-0037] Fortunel, C. , R. Valencia , S. J. Wright , N. C. Garwood , and N. J. Kraft . 2016. “Functional Trait Differences Influence Neighbourhood Interactions in a Hyperdiverse Amazonian Forest.” Ecology Letters 19: 1062–1070. 27358248 10.1111/ele.12642

[ele70028-bib-0038] Garbowski, M. , B. Avera , J. H. Bertram , et al. 2020. “Getting to the Root of Restoration: Considering Root Traits for Improved Restoration Outcomes Under Drought and Competition.” Restoration Ecology 28: 1384–1395.

[ele70028-bib-0039] Gelman, A. , B. Goodrich , J. Gabry , and A. Vehtari . 2019. “R‐Squared for Bayesian Regression Models.” American Statistician 73: 307–309.

[ele70028-bib-0040] Gleason, K. E. , J. B. Bradford , A. Bottero , et al. 2017. “Competition Amplifies Drought Stress in Forests Across Broad Climatic and Compositional Gradients.” Ecosphere 8: e01849.

[ele70028-bib-0041] Gleason, S. M. , C. J. Blackman , A. M. Cook , C. A. Laws , and M. Westoby . 2014. “Whole‐Plant Capacitance, Embolism Resistance and Slow Transpiration Rates all Contribute to Longer Desiccation Times in Woody Angiosperms From Arid and Wet Habitats.” Tree Physiology 34: 275–284. 24550089 10.1093/treephys/tpu001

[ele70028-bib-0042] Goldberg, D. E. 1990. “Components of Resource Competition in Plant Communities.” In Perspectives on Plant Competition, edited by J. B. Grace and D. Tilman , 27–49. Cambridge: Academic Press.

[ele70028-bib-0043] Gourlet‐Fleury, S. , J. M. Guehl , and O. E. Laroussinie . 2004. Ecology and Management of a Neotropical Rainforest: Lessons Drawn From Paracou, a Long‐Term Experimental Research Site in French Guiana. Paris: Elsevier.

[ele70028-bib-0044] Grossiord, C. 2020. “Having the Right Neighbors: How Tree Species Diversity Modulates Drought Impacts on Forests.” New Phytologist 228: 42–49. 30585635 10.1111/nph.15667

[ele70028-bib-0045] Grossiord, C. , A. Gessler , A. Granier , M. Pollastrini , F. Bussotti , and D. Bonal . 2014. “Interspecific Competition Influences the Response of Oak Transpiration to Increasing Drought Stress in a Mixed Mediterranean Forest.” Forest Ecology and Management 318: 54–61.

[ele70028-bib-0046] Grossiord, C. , S. Sevanto , D. Bonal , et al. 2019. “Prolonged Warming and Drought Modify Belowground Interactions for Water Among Coexisting Plants.” Tree Physiology 39: 55–63. 30215810 10.1093/treephys/tpy080

[ele70028-bib-0047] Hafner, B. D. , B. D. Hesse , and T. E. Grams . 2021. “Friendly Neighbours: Hydraulic Redistribution Accounts for One Quarter of Water Used by Neighbouring Drought Stressed Tree Saplings.” Plant, Cell & Environment 44: 1243–1256. 10.1111/pce.13852 32683699

[ele70028-bib-0048] Hérault, B. , B. Bachelot , L. Poorter , et al. 2011. “Functional Traits Shape Ontogenetic Growth Trajectories of Rain Forest Tree Species.” Journal of Ecology 99: 1431–1440.

[ele70028-bib-0049] Higgins, S. I. , T. Conradi , and E. Muhoko . 2023. “Shifts in Vegetation Activity of Terrestrial Ecosystems Attributable to Climate Trends.” Nature Geoscience 16: 147–153.

[ele70028-bib-0050] Hildebrand, M. , M. D. Perles‐Garcia , M. Kunz , W. Härdtle , G. von Oheimb , and A. Fichtner . 2021. “Tree‐Tree Interactions and Crown Complementarity: The Role of Functional Diversity and Branch Traits for Canopy Packing.” Basic and Applied Ecology 50: 217–227.

[ele70028-bib-0051] Hooper, D. U. 1998. “The Role of Complementarity and Competition in Ecosystem Responses to Variation in Plant Diversity.” Ecology 79: 704–719.

[ele70028-bib-0052] Huang, Z. , S. Ran , Y. Fu , et al. 2022. “Functionally Dissimilar Neighbours Increase Tree Water Use Efficiency Through Enhancement of Leaf Phosphorus Concentration.” Journal of Ecology 110: 2179–2189.

[ele70028-bib-0053] Intergovernmental Panel on Climate Change (IPCC) . 2023. Climate Change 2022 – Impacts, Adaptation and Vulnerability. Cambridge: Cambridge University Press.

[ele70028-bib-0054] Jucker, T. , O. Bouriaud , D. Avacaritei , and D. A. Coomes . 2014. “Stabilizing Effects of Diversity on Aboveground Wood Production in Forest Ecosystems: Linking Patterns and Processes.” Ecology Letters 17: 1560–1569. 25308256 10.1111/ele.12382

[ele70028-bib-0055] Jucker, T. , O. Bouriaud , D. Avacaritei , et al. 2014. “Competition for Light and Water Play Contrasting Roles in Driving Diversity–Productivity Relationships in Iberian Forests.” Journal of Ecology 102: 1202–1213.

[ele70028-bib-0056] Jucker, T. , A. C. Sanchez , J. A. Lindsell , H. D. Allen , G. S. Amable , and D. A. Coomes . 2016. “Drivers of Aboveground Wood Production in a Lowland Tropical Forest of West Africa: Teasing Apart the Roles of Tree Density, Tree Diversity, Soil Phosphorus, and Historical Logging.” Ecology and Evolution 6: 4004–4017. 27516859 10.1002/ece3.2175 PMC4875916

[ele70028-bib-0057] Kay, M. 2022. “tidybayes: Tidy Data and Geoms for Bayesian Models.”

[ele70028-bib-0058] Kitajima, K. , and L. Poorter . 2010. “Tissue‐Level Leaf Toughness, but Not Lamina Thickness, Predicts Sapling Leaf Lifespan and Shade Tolerance of Tropical Tree Species.” New Phytologist 186: 708–721. 20298481 10.1111/j.1469-8137.2010.03212.x

[ele70028-bib-0059] Kunstler, G. , D. Falster , D. A. Coomes , et al. 2016. “Plant Functional Traits Have Globally Consistent Effects on Competition.” Nature 529: 204–207. 26700807 10.1038/nature16476

[ele70028-bib-0060] Larocque, G. R. , N. Luckai , S. N. Adhikary , A. Groot , F. W. Bell , and M. Sharma . 2013. “Competition Theory‐Science and Application in Mixed Forest Stands: Review of Experimental and Modelling Methods and Suggestions for Future Research.” Environmental Reviews 21: 71–84.

[ele70028-bib-0061] Lasky, J. R. , M. Uriarte , V. K. Boukili , and R. L. Chazdon . 2014. “Trait‐Mediated Assembly Processes Predict Successional Changes in Community Diversity of Tropical Forests.” Proceedings of the National Academy of Sciences of the United States of America 111: 5616–5621. 24706791 10.1073/pnas.1319342111 PMC3992673

[ele70028-bib-0062] Laurans, M. , B. Hérault , G. Vieilledent , and G. Vincent . 2014. “Vertical Stratification Reduces Competition for Light in Dense Tropical Forests.” Forest Ecology and Management 329: 79–88.

[ele70028-bib-0115] Levionnois, S. , C. Salmon , T. Alméras , et al. 2021. “Anatomies, Vascular Architectures, and Mechanics Underlying The Leaf Size‐Stem Size Spectrum in 42 Neotropical Tree Species.” Journal of Experimental Botany 72: 7957–7969. 34390333 10.1093/jxb/erab379

[ele70028-bib-0064] Levionnois, S. , C. Ziegler , P. Heuret , et al. 2021. “Is Vulnerability Segmentation at the Leaf‐Stem Transition a Drought Resistance Mechanism? A Theoretical Test With a Trait‐Based Model for Neotropical Canopy Tree Species.” Annals of Forest Science 78, no. 4: 1–16.

[ele70028-bib-0065] Liang, J. , T. W. Crowther , N. Picard , et al. 2016. “Positive Biodiversity‐Productivity Relationship Predominant in Global Forests.” Science 354: 196. 10.1126/science.aaf8957 27738143

[ele70028-bib-0066] Liang, J. , M. Zhou , P. C. Tobin , A. D. McGuire , and P. B. Reich . 2015. “Biodiversity Influences Plant Productivity Through Niche‐Efficiency.” Proceedings of the National Academy of Sciences of the United States of America 112: 5738–5743. 25901325 10.1073/pnas.1409853112 PMC4426420

[ele70028-bib-0067] Loreau, M. , and C. de Mazancourt . 2013. “Biodiversity and Ecosystem Stability: A Synthesis of Underlying Mechanisms.” Ecology Letters 16: 106–115. 23346947 10.1111/ele.12073

[ele70028-bib-0068] Luo, Y. , C. L. Ho , B. R. Helliker , and E. Katifori . 2021. “Leaf Water Storage and Robustness to Intermittent Drought: A Spatially Explicit Capacitive Model for Leaf Hydraulics.” Frontiers in Plant Science 12: 2269. 10.3389/fpls.2021.725995 PMC8551678 34721457

[ele70028-bib-0069] Machado, R. , L. Loram‐Lourenço , F. S. Farnese , et al. 2021. “Where Do Leaf Water Leaks Come From? Trade‐Offs Underlying the Variability in Minimum Conductance Across Tropical Savanna Species With Contrasting Growth Strategies.” New Phytologist 229: 1415–1430. 32964437 10.1111/nph.16941

[ele70028-bib-0070] Maréchaux, I. , M. K. Bartlett , L. Sack , et al. 2015. “Drought Tolerance as Predicted by Leaf Water Potential at Turgor Loss Point Varies Strongly Across Species Within an Amazonian Forest.” Functional Ecology 29: 1268–1277.

[ele70028-bib-0071] Maréchaux, I. , L. Saint‐André , M. K. Bartlett , L. Sack , and J. Chave . 2019. “Leaf Drought Tolerance Cannot Be Inferred From Classic Leaf Traits in a Tropical Rainforest.” Journal of Ecology 108: 1030–1045.

[ele70028-bib-0072] Martin‐StPaul, N. K. , S. Delzon , and H. Cochard . 2017. “Plant Resistance to Drought Depends on Timely Stomatal Closure.” Ecology Letters 20: 1437–1447. 28922708 10.1111/ele.12851

[ele70028-bib-0073] Mas, E. , H. Cochard , J. Deluigi , et al. 2024. “Interactions Between Beech and Oak Seedlings Can Modify the Effects of Hotter Droughts and the Onset of Hydraulic Failure.” New Phytologist 241: 1021–1034. 37897156 10.1111/nph.19358

[ele70028-bib-0074] McElreath, R. 2020. Statistical Rethinking: A Bayesian Course With Examples in R and Stan. Boca Raton: CRC Press.

[ele70028-bib-0075] Meng, L. , J. Chambers , C. Koven , et al. 2022. “Soil Moisture Thresholds Explain a Shift From Light‐Limited to Water‐Limited Sap Velocity in the Central Amazon During the 2015–16 El Niño Drought.” Environmental Research Letters 17: 064023.

[ele70028-bib-0076] Michaletz, S. T. , M. D. Weiser , N. G. McDowell , et al. 2016. “The Energetic and Carbon Economic Origins of Leaf Thermoregulation.” Nature Plants 2: 1–9. 10.1038/nplants.2016.129 27548589

[ele70028-bib-0077] Mirabel, A. , B. Hérault , and E. Marcon . 2020. “Diverging Taxonomic and Functional Trajectories Following Disturbance in a Neotropical Forest.” Science of the Total Environment 720: 137397. 32143035 10.1016/j.scitotenv.2020.137397

[ele70028-bib-0078] Moravie, M. A. , J. P. Pascal , and P. Auger . 1997. “Investigating Canopy Regeneration Processes Through Individual‐Based Spatial Models: Application to a Tropical Rain Forest.” Ecological Modelling 104: 241–260.

[ele70028-bib-0079] Moreno, M. , G. Simioni , H. Cochard , et al. 2023. “Functional Diversity Reduces the Risk of Hydraulic Failure in Tree Mixtures Through Hydraulic Disconnection.” *bioRxiv*. 2023.06.09.544345. 10.1101/2023.06.09.544345.

[ele70028-bib-0081] Morin, X. , L. Fahse , M. Scherer‐Lorenzen , and H. Bugmann . 2011. “Tree Species Richness Promotes Productivity in Temperate Forests Through Strong Complementarity Between Species.” Ecology Letters 14: 1211–1219. 21955682 10.1111/j.1461-0248.2011.01691.x

[ele70028-bib-0082] Nemetschek, D. , G. Derroire , E. Marcon , et al. 2024. “Climate Anomalies and Neighbourhood Crowding Interact in Shaping Tree Growth in Old‐Growth and Selectively Logged Tropical Forests.” Journal of Ecology 0: 590–612.

[ele70028-bib-0083] Osnas, J. L. D. , J. W. Lichstein , P. B. Reich , and S. W. Pacala . 2013. “Global Leaf Trait Relationships: Mass, Area, and the Leaf Economics Spectrum.” Science 340: 741–744. 23539179 10.1126/science.1231574

[ele70028-bib-0084] Pardos, M. , M. del Río , H. Pretzsch , et al. 2021. “The Greater Resilience of Mixed Forests to Drought Mainly Depends on Their Composition: Analysis Along a Climate Gradient Across Europe.” Forest Ecology and Management 481: 118687.

[ele70028-bib-0085] Pommerening, A. , and A. J. Sánchez Meador . 2018. “Tamm Review: Tree Interactions Between Myth and Reality.” Forest Ecology and Management 424: 164–176.

[ele70028-bib-0086] Poorter, L. , I. McDonald , A. Alarcón , et al. 2010. “The Importance of Wood Traits and Hydraulic Conductance for the Performance and Life History Strategies of 42 Rainforest Tree Species.” New Phytologist 185: 481–492. 19925555 10.1111/j.1469-8137.2009.03092.x

[ele70028-bib-0087] Prieto, I. , C. Armas , and F. I. Pugnaire . 2012. “Water Release Through Plant Roots: New Insights Into Its Consequences at the Plant and Ecosystem Level.” New Phytologist 193: 830–841. 22250761 10.1111/j.1469-8137.2011.04039.x

[ele70028-bib-0088] R Core Team . 2021. R: A language and environment for statistical computing. Vienna, Austria: R Foundation for Statistical Computing. https://www.R‐project.org/.

[ele70028-bib-0089] Reich, P. B. 2014. “The World‐Wide ‘Fast – Slow’ Plant Economics Spectrum: A Traits Manifesto.” Journal of Ecology 102: 275–301.

[ele70028-bib-0090] Rifai, S. W. , C. A. Girardin , E. Berenguer , et al. 2018. “ENSO Drives Interannual Variation of Forest Woody Growth Across the Tropics.” Philosophical Transactions of the Royal Society, B: Biological Sciences 373: 20170410. 10.1098/rstb.2017.0410 PMC6178425 30297475

[ele70028-bib-0091] Rodriguez‐Dominguez, C. M. , T. N. Buckley , G. Egea , et al. 2016. “Most Stomatal Closure in Woody Species Under Moderate Drought Can Be Explained by Stomatal Responses to Leaf Turgor.” Plant, Cell & Environment 39: 2014–2026. 10.1111/pce.12774 27255698

[ele70028-bib-0092] RStudio Team . 2020. RStudio: Integrated Development Environment for R. Boston, MA: RStudio, PBC.

[ele70028-bib-0093] Scheidegger, Y. , M. Saurer , M. Bahn , and R. Siegwolf . 2000. “Linking Stable Oxygen and Carbon Isotopes With Stomatal Conductance and Photosynthetic Capacity: A Conceptual Model.” Oecologia 125: 350–357. 28547329 10.1007/s004420000466

[ele70028-bib-0094] Schnabel, F. , K. E. Barry , S. Eckhardt , et al. 2024. “Neighbourhood Species Richness and Drought‐Tolerance Traits Modulate Tree Growth and δ^13^C Responses to Drought.” Plant Biology 26: 330–345. 38196270 10.1111/plb.13611

[ele70028-bib-0095] Schnabel, F. , X. Liu , M. Kunz , et al. 2021. “Species Richness Stabilizes Productivity via Asynchrony and Drought‐Tolerance Diversity in a Large‐Scale Tree Biodiversity Experiment.” Science Advances 7: 1643. 10.1126/sciadv.abk1643 PMC8682986 34919425

[ele70028-bib-0096] Stahl, C. , B. Hérault , V. Rossi , B. Burban , C. Bréchet , and D. Bonal . 2013. “Depth of Soil Water Uptake by Tropical Rainforest Trees During Dry Periods: Does Tree Dimension Matter?” Oecologia 173: 1191–1201. 23852028 10.1007/s00442-013-2724-6

[ele70028-bib-0097] Stan Development Team . 2022. “CmdStanR: the R interface to CmdStan.”

[ele70028-bib-0098] Tymen, B. , G. Vincent , E. A. Courtois , et al. 2017. “Quantifying Micro‐Environmental Variation in Tropical Rainforest Understory at Landscape Scale by Combining Airborne LiDAR Scanning and a Sensor Network.” Annals of Forest Science 74: 32.

[ele70028-bib-0099] Uriarte, M. , N. G. Swenson , R. L. Chazdon , et al. 2010. “Trait Similarity, Shared Ancestry and the Structure of Neighbourhood Interactions in a Subtropical Wet Forest: Implications for Community Assembly.” Ecology Letters 13: 1503–1514. 21054732 10.1111/j.1461-0248.2010.01541.x

[ele70028-bib-0100] Vile, D. , É. Garnier , B. Shipley , et al. 2005. “Specific Leaf Area and Dry Matter Content Estimate Thickness in Laminar Leaves.” Annals of Botany 96: 1129–1136. 16159941 10.1093/aob/mci264 PMC4247101

[ele70028-bib-0101] Vleminckx, J. , C. Fortunel , O. Valverde‐Barrantes , et al. 2021. “Resolving Whole‐Plant Economics From Leaf, Stem and Root Traits of 1467 Amazonian Tree Species.” Oikos 130: 1193–1208.

[ele70028-bib-0102] Volaire, F. 2018. “A Unified Framework of Plant Adaptive Strategies to Drought: Crossing Scales and Disciplines.” Global Change Biology 24: 2929–2938. 29350812 10.1111/gcb.14062

[ele70028-bib-0103] Wagner, F. , V. Rossi , C. Baraloto , D. Bonal , C. Stahl , and B. Hérault . 2014. “Are Commonly Measured Functional Traits Involved in Tropical Tree Responses to Climate?” International Journal of Ecology 2014: 389409.

[ele70028-bib-0104] Wagner, F. H. , B. Hérault , D. Bonal , et al. 2016. “Climate Seasonality Limits Leaf Carbon Assimilation and Wood Productivity in Tropical Forests.” Biogeosciences 13: 2537–2562.

[ele70028-bib-0105] Westoby, M. , D. S. Falster , A. T. Moles , P. A. Vesk , and I. J. Wright . 2002. “Plant Ecological Strategies: Some Leading Dimensions of Variation Between Species.” Annual Review of Ecology and Systematics 33: 125–159.

[ele70028-bib-0106] Wickham, H. , M. Averick , J. Bryan , et al. 2019. “Welcome to the Tidyverse.” Journal of Open Source Software 4: 1686.

[ele70028-bib-0107] Wright, A. J. 2024. “Plant–Plant Interactions Can Mitigate (Or Exacerbate) hot Drought Impacts.” New Phytologist 241: 955–957. 38087824 10.1111/nph.19473

[ele70028-bib-0108] Wright, I. J. , N. Dong , V. Maire , et al. 2017. “Global Climatic Drivers of Leaf Size.” Science 357: 917–921. 28860384 10.1126/science.aal4760

[ele70028-bib-0109] Wright, I. J. , P. B. Reich , M. Westoby , et al. 2004. “The Worldwide Leaf Economics Spectrum.” Nature 428: 821–827. 15103368 10.1038/nature02403

[ele70028-bib-0110] Yu, W. , G. Albert , B. Rosenbaum , et al. 2024. “Systematic Distributions of Interaction Strengths Across Tree Interaction Networks Yield Positive Diversity–Productivity Relationships.” Ecology Letters 27: e14338. 38030225 10.1111/ele.14338

[ele70028-bib-0111] Zhang, S. , D. Landuyt , K. Verheyen , and P. De Frenne . 2022. “Tree Species Mixing Can Amplify Microclimate Offsets in Young Forest Plantations.” Journal of Applied Ecology 59: 1428–1439.

[ele70028-bib-0112] Ziegler, C. , H. Cochard , C. Stahl , et al. 2024. “Residual Water Losses Mediate the Trade‐Off Between Growth and Drought Survival Across Saplings of 12 Tropical Rainforest Tree Species With Contrasting Hydraulic Strategies.” Journal of Experimental Botany 75: 4128–4147. 38613495 10.1093/jxb/erae159

[ele70028-bib-0113] Ziegler, C. , S. Coste , C. Stahl , et al. 2019. “Large Hydraulic Safety Margins Protect Neotropical Canopy Rainforest Tree Species Against Hydraulic Failure During Drought.” Annals of Forest Science 76: 115.

