# Supplementary material for: Love Thy Neighbour? Tropical Tree Growth and Its Response to Climate Anomalies Is Mediated by Neighbourhood Hierarchy and Dissimilarity in Carbon‐ and Water‐Related Traits
Source: Ecol Lett. 2025 Apr 8;28(4):e70028. doi: 10.1111/ele.70028 (PMC11977451; doi:10.1111/ele.70028)
Supplement: Supplementary file 1 — Figure S1. Adapted figure from Nemetschek et al. (2024): Mean standardised climate anomalies at Paracou for the two‐year census intervals over the study period. Figure S2. Absolute values of mean monthly climate indices in comparison with their respective 30‐year monthly mean for the 1991–2021 period. Figure S3. Correlation matrix showing pairwise Pearson correlation coefficients between all neighbourhood indices. Figure S4. Standardised regression coefficients of community‐level parameter estimates from NIh models. Figure S5. Standardised regression coefficients of community‐level parameter estimates from NId models. Figure S6. Overview of species‐level responses to NIh and NId that clearly deviate from the community‐level response. Methods S1. Corrections of tree inventory data. Methods S2. Gapfilling of missing species information. Methods S3. Calculation of climate anomalies. Methods S4. Additional information on neighbourhood indices. Methods S5. Transformation of response variable and model covariates. Methods S6. Full model equation. Methods S7. Information on model stability. Methods S8. Spatial autocorrelation check. [file ELE-28-0-s001.zip › ele70028-sup-0006-FigureS6.html]

 


# 

## x

|  | NId | | | | | | | | | | | | | | | | | | | | | | | | | | | NIh | | | | | | | | | | | | | | | | | | | | | | | | | | |
| --- | --- | --- | --- | --- | --- | --- | --- | --- | --- | --- | --- | --- | --- | --- | --- | --- | --- | --- | --- | --- | --- | --- | --- | --- | --- | --- | --- | --- | --- | --- | --- | --- | --- | --- | --- | --- | --- | --- | --- | --- | --- | --- | --- | --- | --- | --- | --- | --- | --- | --- | --- | --- | --- | --- |
|  | LA | | | LSWC | | | Lthick | | | Ltough | | | SLA | | | WSG | | | d13C | | | gmin | | | tlp | | | LA | | | LSWC | | | Lthick | | | Ltough | | | SLA | | | WSG | | | d13C | | | gmin | | | tlp | | |
|  | CWD | Tmax | VPD | CWD | Tmax | VPD | CWD | Tmax | VPD | CWD | Tmax | VPD | CWD | Tmax | VPD | CWD | Tmax | VPD | CWD | Tmax | VPD | CWD | Tmax | VPD | CWD | Tmax | VPD | CWD | Tmax | VPD | CWD | Tmax | VPD | CWD | Tmax | VPD | CWD | Tmax | VPD | CWD | Tmax | VPD | CWD | Tmax | VPD | CWD | Tmax | VPD | CWD | Tmax | VPD | CWD | Tmax | VPD |
| Cecropia\_obtusa | + | + | + | + | + | + | + | + | + | + | + | + | + | + | + | + | + | + | + | + | + | + | + | + | + | + | + | + | + | + | + | + | + | + | + | + | - | - | - | - | - | - | - | - | - | - | - | - | + | + | + | + | + | + |
| Eperua\_falcata | + | + | + | + | + | + | + | + | + | + | + | + | + | + | + | + | + | + | + | + | + | + | + | + | + | + | + | - | - | - |  |  |  | - | - | - | - | - | - | + | + | + | - | - | - | + | + | + | - | - | - | - | - | - |
| Inga\_alba | + | + | + | + | + | + | + | + | + | + | + | + | + | + | + | + | + | + | + | + | + | + | + | + |  |  |  | - | - | - | - | - | - | - | - | - | - | - | - | + | + | + | - | - | - | + | + | + | - | - | - |  |  |  |
| Miconia\_acuminata | + | + | + | + | + | + | + | + | + | + | + | + |  |  | + | + | + | + | + | + | + | + | + | + | + | + | + | - | - | - | - |  | - | - | - | - | - | - | - | + | + | + | - | - | - | + | + | + | - | - | - |  |  |  |
| Sterculia\_pruriens | + | + | + | + | + | + | + | + | + |  |  |  | + | + | + | + | + | + | + | + | + | + | + | + |  | + |  | - | - | - | + | + | + | - | - | - | - | - | - | + | + | + | - | - | - | + | + | + | - |  | - | + | + | + |
| Tapirira\_obtusa | + | + | + | + | + | + | + | + | + | + | + | + | + | + | + | + | + | + | + | + | + |  |  |  | + | + | + | - | - | - | - | - | - | - | - | - | - | - | - | + | + | + | - | - | - | + | + | + | - | - | - |  |  |  |
| Tapirira\_guianensis | + | + | + | + | + | + | + | + | + | + | + | + | + | + | + | + | + | + | + | + | + | + | + | + |  | + |  | - | - | - | - | - | - | - | - | - | - | - | - | + | + | + |  |  |  | + | + | + | - | - | - |  |  |  |
| Abarema\_jupunba | + | + | + | + | + | + | + | + | + | + | + | + | + | + | + | + | + | + | + | + | + | + | + | + |  |  |  | - | - | - | - | - | - | - | - | - | - | - | - | + | + | + | - |  |  |  |  |  | - | - | - |  |  |  |
| Jacaranda\_copaia | + | + | + | + |  |  |  |  |  | + | + | + | + | + | + | + | + | + | - | - | - | + |  | + |  |  |  | - | - | - |  |  |  | - | - | - | - | - | - | + | + | + | - | - | - | + | + | + | - | - | - |  |  |  |
| Laetia\_procera | + | + | + | + | + | + | + | + | + |  |  |  | + | + | + | + | + | + |  |  |  | + | + | + |  |  |  | - | - | - | - | - | - | - | - | - | - |  |  | + | + | + | - |  | - |  |  |  | - | - | - |  |  |  |
| Bellucia\_arborescens | + | + | + |  |  |  | + | + | + |  |  |  | + | + | + | + | + | + |  |  |  | + | + | + | + | + |  | - | - | - | - | - | - | - | - | - |  |  |  | + | + | + |  |  |  | + | + | + | - | - | - |  |  |  |
| Didymopanax\_decaphyllus | + | + | + | + | + |  |  |  |  |  |  |  | - |  |  | + | + | + |  |  |  | + | + | + | + | + | + | - | - | - |  |  | - |  |  | - | - | - | - | + |  |  | - | - | - | + | + | + | - | - | - |  |  | - |
| Xylopia\_nitida | + | + | + | + | + | + |  |  |  |  |  |  |  |  |  | + | + | + | + | + | + | + | + | + |  |  |  | - | - | - |  |  |  | - | - |  | - | - | - | + | + | + |  |  |  | + | + | + | - | - | - |  |  |  |
| Pradosia\_cochlearia | + | + | + |  |  |  | + | + | + | + | + | + |  |  |  | + | + | + | + | + | + | + | + | + |  |  |  | - | - | - |  |  |  | - | - | - | - |  | - |  |  |  |  |  |  |  |  |  | - | - | - |  |  |  |
| Tachigali\_melinonii |  |  |  | + | + | + | + | + | + | + | + | + | + | + | + |  |  |  | + | + | + |  |  |  |  |  |  | - |  |  | - | - | - | - | - | - | - |  |  | + | + | + |  |  |  | + | + | + |  |  |  |  |  |  |
| Eschweilera\_sagotiana | + | + | + |  |  |  | + | + | + | + | + | + |  | + |  | + | + | + | + | + | + |  |  |  |  |  |  |  |  |  |  |  |  | + | + | + | + | + | + | - | - | - |  |  |  | - | - | - |  |  |  |  |  |  |
| Eperua\_grandiflora | + | + | + |  |  |  |  |  |  |  |  |  |  |  |  | + | + | + | + | + | + | + | + | + | + | + | + | - | - | - | - | - | - |  |  |  |  |  |  |  |  |  |  |  |  |  |  |  | - | - | - | - | - | - |
| Carapa\_surinamensis | + | + | + | + | + | + | + | + | + |  |  |  | + | + | + |  |  |  | + | + | + |  |  |  | - | - |  |  |  |  |  |  |  |  |  |  |  |  |  | - | - | - | - |  |  |  |  |  |  |  |  |  |  |  |
| Protium\_opacum | + | + | + |  |  |  |  |  |  |  |  |  | - | - | - |  |  |  | + | + | + |  |  |  |  |  |  | - | - | - |  |  |  | + | + | + |  |  |  | - | - | - |  |  |  | - | - | - |  |  |  |  |  |  |
| Qualea\_rosea | + | + | + |  |  |  |  |  |  |  |  |  | + | + | + | + | + | + | + | + | + |  |  |  |  |  |  | - | - | - |  |  |  |  |  |  |  |  |  |  |  |  | + | + | + | + | + | + |  |  |  |  |  |  |
| Catostemma\_fragrans |  |  |  | + | + | + |  |  |  |  |  |  |  |  |  |  |  |  | + | + | + |  |  |  |  |  |  | - | - | - |  |  |  |  |  |  |  |  |  |  |  |  | - | - | - |  |  |  | - | - | - | - | - | - |
| Protium\_subserratum | + | + | + |  |  |  |  |  |  |  |  |  |  |  |  |  |  |  | + | + | + | + | + | + |  |  |  | - | - | - |  |  |  | - | - |  |  |  |  |  |  |  |  |  |  |  |  |  | - | - | - |  |  |  |
| Mouriri\_crassifolia |  |  |  |  |  |  | - |  | - | - |  | - | - | - | - | - | - | - |  |  |  |  |  |  |  |  |  |  |  |  |  |  |  | - | - | - | - | - | - |  |  |  | - |  |  |  |  |  |  |  |  |  |  |  |
| Inga\_stipularis | + | + | + | + | + | + |  |  |  |  |  |  |  |  |  |  |  |  |  |  |  |  |  |  |  |  |  |  |  |  |  |  |  |  |  | - | - | - | - | + | + | + | - | - | - |  |  |  |  |  |  |  |  |  |
| Mabea\_piriri |  |  |  |  |  |  |  |  |  |  |  |  | - | - | - |  | - |  | + | + | + |  |  |  |  |  |  |  |  |  |  |  |  | + | + | + |  |  |  |  |  |  |  |  |  | - | - | - | + | + | + |  |  |  |
| Moronobea\_coccinea |  |  |  |  |  |  |  |  |  |  |  |  | + | + | + | + |  |  |  |  |  |  |  |  |  |  |  |  |  |  |  |  |  | - | - | - | - | - | - | + | + | + | - | - | - |  |  |  |  |  |  |  |  |  |
| Drypetes\_variabilis | + | + | + | + | + | + | - |  | - |  |  |  |  |  |  |  |  |  |  |  |  | + |  |  |  |  |  |  |  |  |  |  |  |  |  |  |  |  |  | + | + | + | - | - | - |  |  |  |  |  |  |  |  |  |
| Oxandra\_asbeckii | - | - |  |  |  |  | + | + | + |  |  |  | + | + | + | - | - | - |  |  |  |  |  |  |  |  |  |  |  |  |  |  |  |  |  |  | - |  |  |  |  |  | - | - | - |  |  |  |  |  |  |  |  |  |
| Hymenopus\_heteromorphus |  |  |  |  |  |  | + | + | + |  |  |  |  |  |  | - | - | - | - | - | - |  |  |  |  |  |  |  |  |  |  |  |  |  |  |  |  |  |  |  |  |  | - | - | - | + | + | + |  |  |  |  |  |  |
| Chrysophyllum\_prieurii |  |  |  |  |  |  |  |  |  |  |  |  | - |  |  | - | - | - |  |  |  |  |  |  |  |  |  |  |  |  |  |  |  | - | - | - | - | - | - |  |  |  | - |  |  | + | + | + |  |  |  |  |  |  |
| Sextonia\_rubra |  |  |  |  |  |  |  |  |  |  |  |  | - |  | - | + | + | + |  |  |  |  |  |  |  |  |  |  |  |  |  |  |  |  |  |  |  |  |  | + | + | + | - | - | - |  |  |  | - |  | - | - |  |  |
| Licania\_membranacea |  |  |  | + | + | + |  |  |  |  |  |  |  |  |  |  |  |  |  |  |  |  |  |  | + | + | + |  |  |  | - | - | - | - |  |  |  |  |  |  |  |  |  |  |  |  |  |  |  |  |  | - | - | - |
| Goupia\_glabra |  |  |  | + | + | + |  |  |  |  |  |  |  |  |  | + |  |  |  |  |  |  |  |  |  |  |  |  |  |  | - | - | - |  | - |  |  |  |  |  |  |  |  |  |  | + | + | + |  |  |  |  |  |  |
| Iryanthera\_sagotiana |  |  |  | + | + | + |  |  |  |  |  |  |  |  |  | + |  | + |  |  |  |  |  |  |  |  |  | - | - |  |  |  |  |  |  |  |  |  |  |  |  |  | - | - | - |  |  |  |  |  |  |  |  |  |
| Lecythis\_persistens |  |  |  | + |  |  |  |  |  |  |  |  |  |  |  |  |  |  |  |  |  |  |  |  |  |  |  |  |  |  |  |  |  |  |  |  |  |  |  | - | - | - | - | - | - | - | - | - |  |  |  |  |  |  |
| Eschweilera\_coriacea |  |  |  |  |  |  | + | + | + |  |  |  | + | + | + |  |  |  |  |  |  |  |  |  |  |  |  |  |  |  |  |  |  |  |  |  | - |  | - |  |  |  | - |  | - |  |  |  |  |  |  |  |  |  |
| Recordoxylon\_speciosum |  |  |  |  |  |  |  |  |  |  |  |  |  |  |  | + | + | + |  |  |  | - | - | - |  |  |  |  |  |  |  |  |  |  |  |  |  |  |  |  |  |  | + | + | + |  |  |  |  |  |  |  |  |  |
| Dicorynia\_guianensis | + | + | + |  |  |  | + |  |  |  |  |  |  |  |  | + | + | + |  |  |  |  |  |  |  |  |  | - |  | - |  |  |  |  |  |  |  |  |  |  |  |  |  |  |  |  |  |  |  |  |  |  |  |  |
| Symphonia\_globulifera |  |  |  |  |  |  |  |  |  |  |  |  | - | - | - |  |  |  | + | + | + |  |  |  |  |  |  |  |  |  |  |  |  |  |  |  |  |  |  |  |  |  |  |  |  | + | + | + |  |  |  |  |  |  |
| Virola\_michelii |  |  |  |  |  |  |  |  |  |  |  |  |  |  |  |  |  |  |  |  |  |  |  |  |  |  |  | - | - | - |  |  |  |  |  |  | - | - | - |  |  |  |  |  |  | + |  |  | - | - |  |  |  |  |
| Poraqueiba\_guianensis |  |  |  |  |  |  | + |  |  |  |  |  |  |  |  |  |  |  |  |  |  | + | + | + |  |  |  |  |  |  | - | - |  |  |  |  |  |  |  |  |  |  |  |  |  |  |  |  | - | - |  |  |  |  |
| Theobroma\_guianense |  |  |  |  |  |  |  |  |  |  |  |  |  |  |  |  |  |  |  |  |  | + | + | + |  |  |  |  |  |  |  |  |  | - | - |  |  |  |  |  |  |  |  |  |  |  |  |  | - | - | - |  |  |  |
| Eschweilera\_congestiflora |  |  |  |  |  |  |  |  |  |  |  |  |  |  |  |  |  |  |  |  |  | + | + | + |  |  |  |  |  |  |  |  |  |  |  |  |  |  |  |  |  |  |  |  |  |  |  |  | - | - | - |  |  |  |
| Caryocar\_glabrum | - | - | - | + | + | + |  |  |  |  |  |  |  |  |  |  |  |  |  |  |  |  |  |  |  |  |  |  |  |  |  |  |  |  |  |  |  |  |  |  |  |  |  |  |  |  |  |  |  |  |  |  |  |  |
| Licania\_alba |  |  |  | + |  |  |  |  |  |  |  |  |  |  |  | - |  | - |  |  |  |  |  |  | + | + | + |  |  |  |  |  |  |  |  |  |  |  |  |  |  |  |  |  |  |  |  |  |  |  |  |  |  |  |
| Protium\_stevensonii |  |  |  | + | + | + |  |  |  |  |  |  |  |  |  |  |  |  |  |  |  |  |  |  |  |  |  |  |  |  | - | - | - |  |  |  |  |  |  |  |  |  |  |  |  |  |  |  |  |  |  |  |  |  |
| Homalolepis\_cedron |  |  |  |  |  |  |  |  |  |  |  |  |  |  |  |  |  |  | - | - | - |  |  |  |  |  |  |  |  |  |  |  |  |  |  |  |  |  |  |  |  |  |  |  |  | - | - | - |  |  |  |  |  |  |
| Garcinia\_madruno |  |  |  |  |  |  |  |  |  |  |  |  |  |  |  |  |  |  |  |  |  |  |  |  |  |  |  |  |  |  |  |  |  |  |  |  |  |  |  |  |  |  | - | - | - |  |  |  | - | - | - |  |  |  |
| Dendrobangia\_boliviana | + |  |  |  |  |  |  |  |  |  |  |  |  |  |  | + |  |  |  |  |  |  |  |  |  |  |  |  |  |  |  |  |  |  |  |  |  |  |  |  |  |  | - | - | - |  |  |  |  |  |  |  |  |  |
| Monteverdia\_oblongata |  |  |  |  | + | + |  |  |  |  |  |  |  |  |  |  |  |  |  |  |  |  |  |  |  |  |  |  |  |  |  |  |  |  |  |  |  |  |  | + | + | + |  |  |  |  |  |  |  |  |  |  |  |  |
| Licania\_canescens |  |  |  |  |  |  |  |  |  |  |  |  |  |  |  | - |  |  |  |  |  |  |  |  |  |  |  |  |  |  |  |  |  |  |  |  | - |  |  |  |  |  | - | - | - |  |  |  |  |  |  |  |  |  |
| Cordia\_sagotii |  |  |  |  |  | + |  |  |  |  |  |  |  |  |  |  |  |  |  |  |  |  |  |  |  |  |  |  |  |  |  |  |  |  |  |  |  |  |  |  |  |  | - | - | - |  |  |  |  |  |  |  |  |  |
| Chrysophyllum\_sanguinolentum |  |  |  |  |  |  |  |  |  |  |  |  |  |  |  |  |  |  |  |  |  | - | - | - |  |  |  |  |  |  |  |  |  |  |  |  |  |  |  |  |  |  |  |  |  |  |  |  |  |  |  |  |  |  |
| Eschweilera\_decolorans |  |  |  |  |  |  |  |  |  |  |  |  |  |  |  |  |  |  |  |  |  | + |  | + |  |  |  |  |  |  |  |  |  |  |  |  |  |  |  |  |  |  |  |  |  |  |  |  |  |  | - |  |  |  |
| Conceveiba\_guianensis | + | + | + |  |  |  |  |  |  |  |  |  |  |  |  |  |  |  |  |  |  |  |  |  |  |  |  |  |  |  |  |  |  |  |  |  |  |  |  |  |  |  |  |  |  |  |  |  |  |  |  |  |  |  |
| Symphonia\_sp.1 |  |  |  |  |  |  |  |  |  |  |  |  |  |  |  |  |  |  |  |  |  |  |  |  | - | - |  |  |  |  |  |  |  |  |  |  |  |  |  |  |  |  |  |  |  |  |  | - |  |  |  |  |  |  |
| Gustavia\_hexapetala |  |  |  |  |  |  |  |  |  |  |  |  |  |  |  |  |  |  | + | + | + |  |  |  |  |  |  |  |  |  |  |  |  |  |  |  |  |  |  |  |  |  |  |  |  |  |  |  |  |  |  |  |  |  |
| Pogonophora\_schomburgkiana |  |  |  |  |  |  |  |  |  |  |  |  |  |  |  |  |  |  |  |  |  |  |  |  |  |  |  |  |  |  |  |  |  |  |  |  |  |  |  |  |  |  |  |  |  |  |  |  | - | - | - |  |  |  |
| Bocoa\_prouacensis |  |  |  |  |  |  |  |  |  |  |  |  |  |  |  |  |  |  |  |  |  |  |  |  |  |  |  | + | + | + |  |  |  |  |  |  |  |  |  |  |  |  |  |  |  |  |  |  |  |  |  |  |  |  |
| Inga\_loubryana | + |  |  |  |  |  |  |  |  |  |  |  |  |  |  |  |  |  |  |  |  | + |  |  |  |  |  |  |  |  |  |  |  |  |  |  |  |  |  |  |  |  |  |  |  |  |  |  |  |  |  |  |  |  |
| Vouacapoua\_americana |  |  |  |  |  |  |  |  |  |  |  |  |  |  |  |  |  |  |  |  |  |  |  |  |  |  |  |  |  |  |  |  |  |  |  |  |  |  |  |  |  |  |  |  |  |  |  |  |  |  |  |  | - | - |
| Hevea\_guianensis |  |  |  |  |  |  |  |  |  |  |  |  |  |  |  |  |  |  |  |  |  |  |  |  |  |  |  |  |  |  |  |  |  |  |  |  |  |  |  |  |  |  | - |  |  |  |  |  |  |  |  |  |  |  |

Figure S6. Species-level responses to NI (either NIh or NId) that clearly deviate from the community level response (“+” for clear positive deviation in green, “-” for clear negative deviation in yellow), for each of the 54 models. A clear deviation is defined as a case when the 95%-HPDI interval of the species-level parameter does not overlap the 95%-HPDI interval of the community-level parameter. Only the 62 species that have a clear species-level response to at least one of the model covariates are presented, in decreasing order of the number of model parameters for which they have a clear species-level deviation. We present only the species-level response to NI (𝛽4s) as the species-level response to the NI\*Climate interaction (𝛽6s) rarely deviated from the community-level response. We indeed found that none of the models had more than 10% of the species (9 species) clearly deviating from the community-level response for the NI\*Climate interaction parameter, when 26 out of 54 models had more than 10% of the species clearly deviating from the community-level response for the NId parameter, and 24 out of 54 models for the NIh parameter.
