# Supplementary material for: Love Thy Neighbour? Tropical Tree Growth and Its Response to Climate Anomalies Is Mediated by Neighbourhood Hierarchy and Dissimilarity in Carbon‐ and Water‐Related Traits
Source: Ecol Lett. 2025 Apr 8;28(4):e70028. doi: 10.1111/ele.70028 (PMC11977451; doi:10.1111/ele.70028)
Supplement: Supplementary file 1 — Figure S1. Adapted figure from Nemetschek et al. (2024): Mean standardised climate anomalies at Paracou for the two‐year census intervals over the study period. Figure S2. Absolute values of mean monthly climate indices in comparison with their respective 30‐year monthly mean for the 1991–2021 period. Figure S3. Correlation matrix showing pairwise Pearson correlation coefficients between all neighbourhood indices. Figure S4. Standardised regression coefficients of community‐level parameter estimates from NIh models. Figure S5. Standardised regression coefficients of community‐level parameter estimates from NId models. Figure S6. Overview of species‐level responses to NIh and NId that clearly deviate from the community‐level response. Methods S1. Corrections of tree inventory data. Methods S2. Gapfilling of missing species information. Methods S3. Calculation of climate anomalies. Methods S4. Additional information on neighbourhood indices. Methods S5. Transformation of response variable and model covariates. Methods S6. Full model equation. Methods S7. Information on model stability. Methods S8. Spatial autocorrelation check. [file ELE-28-0-s001.zip › ele70028-sup-0007-Supinfo01.pdf]

Supplementary Figures

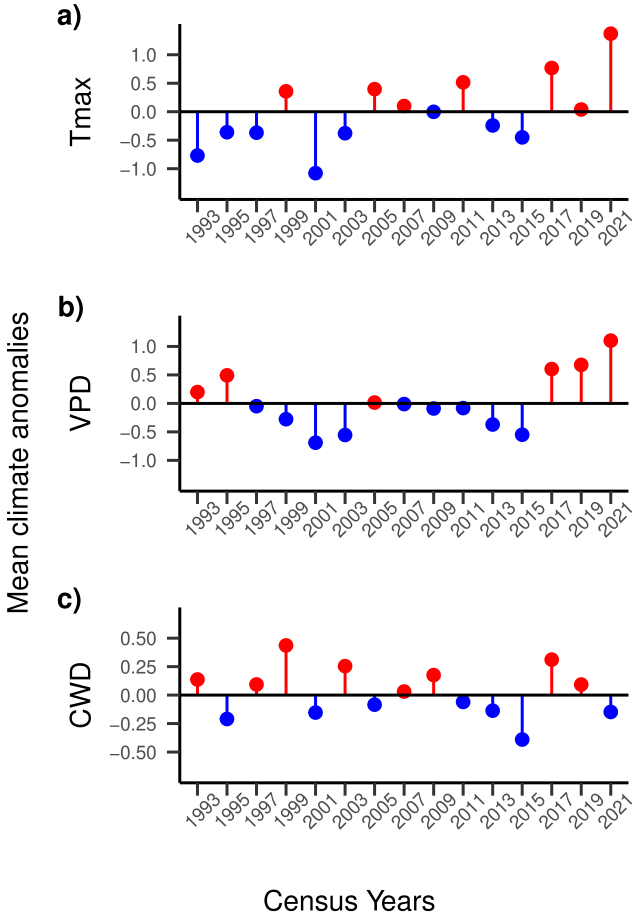

Figure **S1.** Adapted figure from Nemetschek *et al.* (2024): Mean standardised climate anomalies at Paracou for the two-year census intervals over the study period for a) maximum temperature, b) vapour pressure deficit and c) climatic water deficit. Red and blue lines and circles represent positive and negative anomalies respectively.

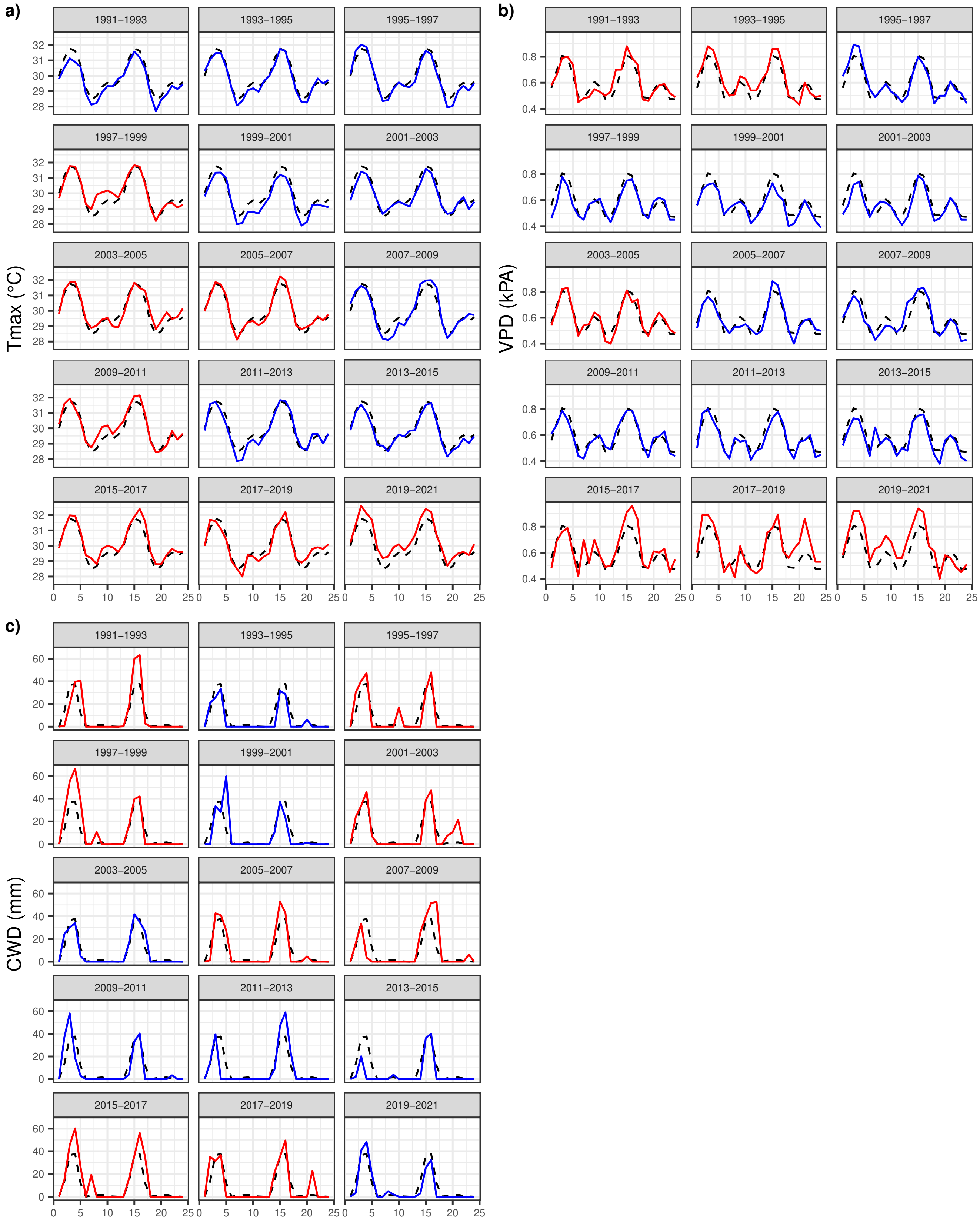

Figure **S2.** Absolute values of mean monthly climate indices (solid lines) in comparison to their respective 30-year monthly mean for the 1991-2021 period (dashed lines) for a) maximum temperature, b) vapour pressure deficit and c) climatic water deficit and each of the 15 census intervals. Red and blue lines correspond to Fig. S1 and respectively indicate census intervals with overall higher positive or negative anomalies in these climate indices.

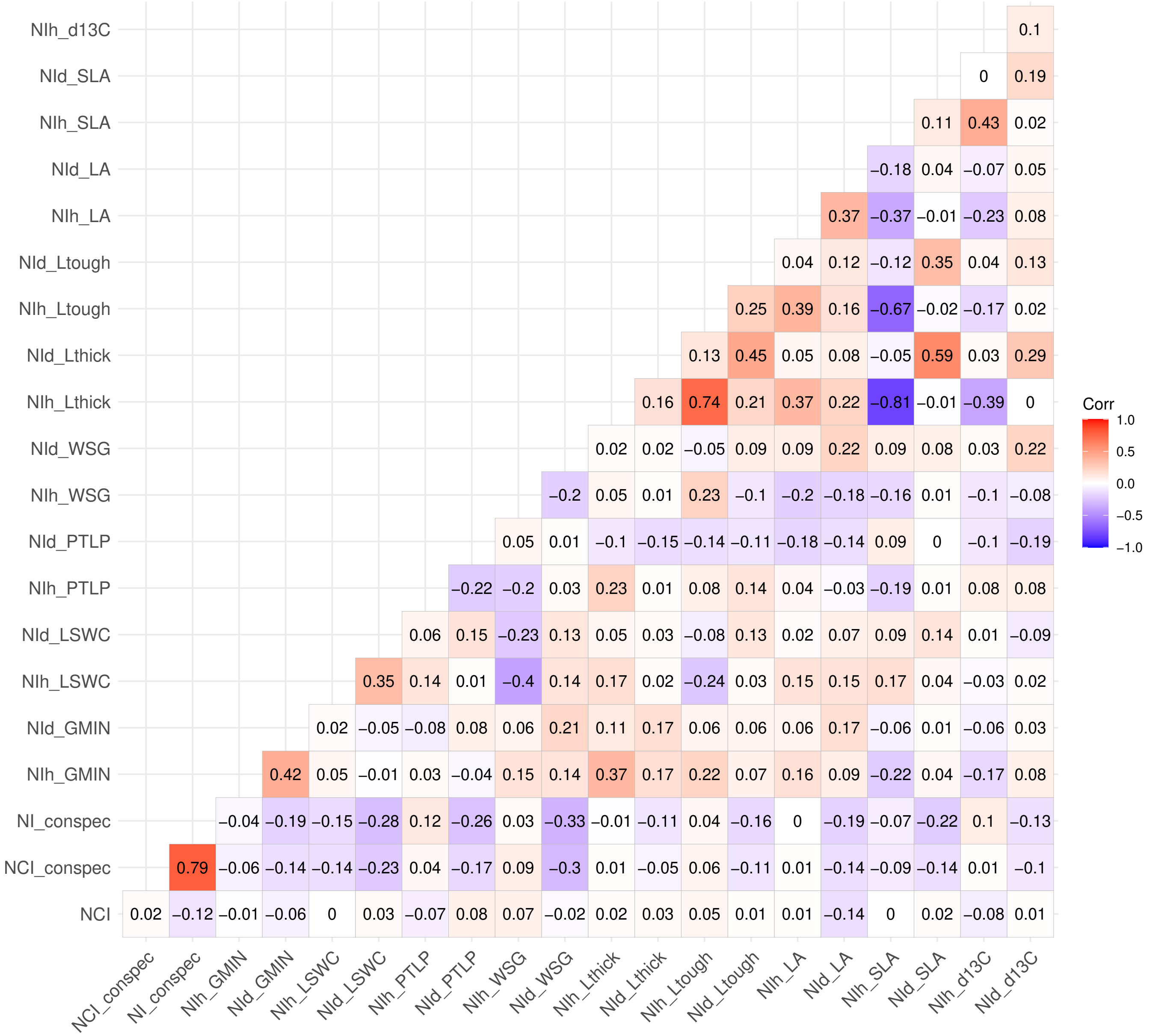

Figure **S3.** Correlation matrix showing pairwise Pearson correlations coefficients between all neighbourhood indices (log transformed). NId_Traitname and NIh_Traitname refer to neighbourhood indices integrating neighbourhood trait dissimilarities and hierarchies for the respective trait. NCI refers to the neighbourhood index capturing neighbourhood crowding (i.e. densities). NCI_conspecific and NI_conspecific refer to additionally calculated neighbourhood matrices respectively representing conspecific densities (calculated as NCI but only taking into account conspecific neighbours) and the proportion of conspecifics to total NCI. Blue and red colouring respectively denotes positive and negative correlations. The intensity of the colours reflects the strength of the respective correlation.

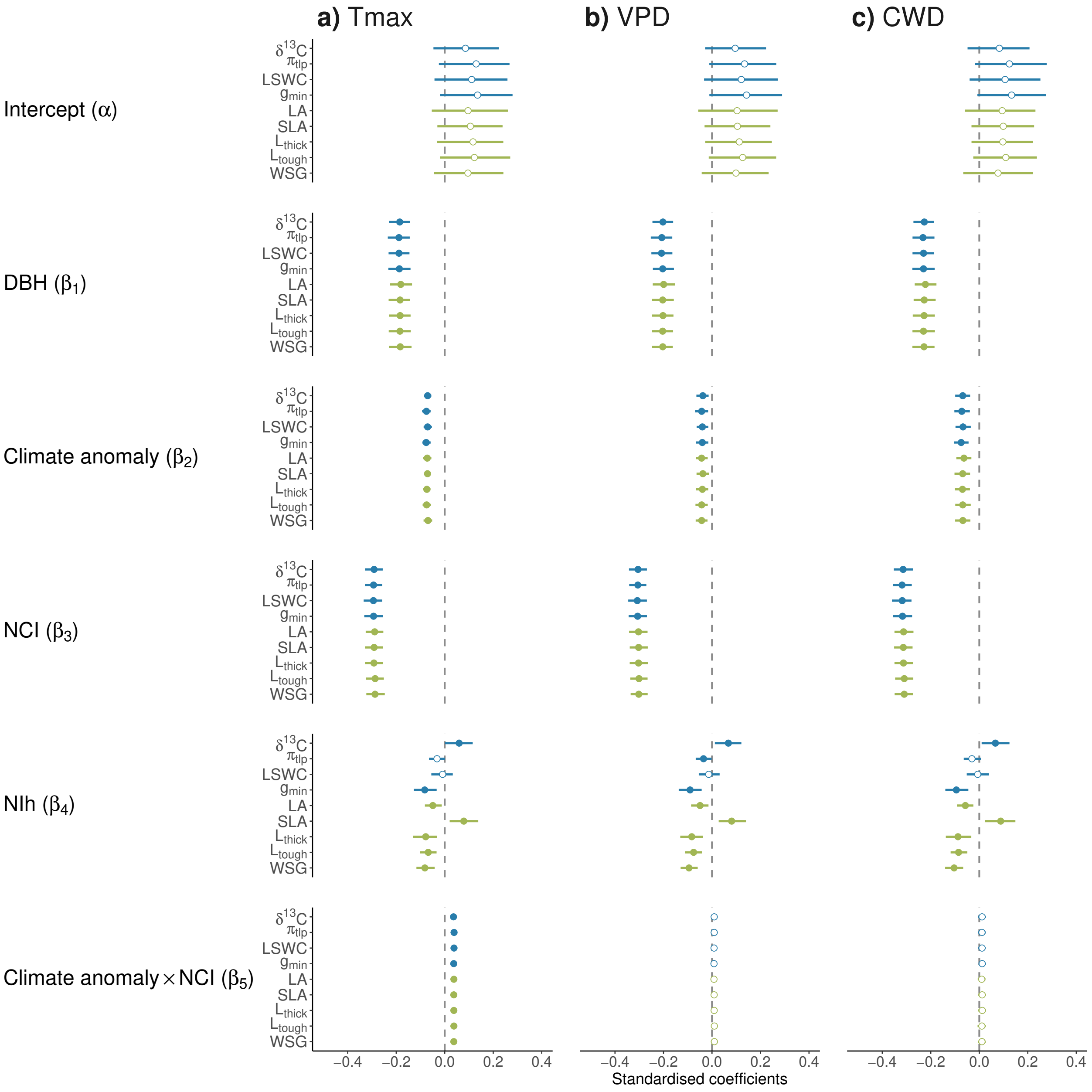

Figure **S4.** Community-level parameter estimates from the 27 NIh models showing intrinsic AGR (Intercept, α) and community level AGR response to tree size (DBH, β_1), climate anomaly (β_2), neutral neighbourhood crowding (NCI, β_3), the hierarchical neighbourhood index (NIh, β_4) and the interactions between climate anomaly and neutral neighbourhood crowding (Climate anomaly×NCI, β_5). Parameter estimates of the response to NIh (β_4) and the interactions between climate anomaly and NIh (β_6) are presented in the main text (Fig. 2 and 3. Standardised coefficients are shown separately for the three climate models: a) Tmax, b) VPD and c) CWD models and each of the nine trait models: carbon $\delta^{13}C$ isotope composition, water potential at turgor loss point (π_{tlp}), leaf saturated water content (LSWC), minimum conductance (g_{min}), leaf area (LA), specific leaf area (SLA), leaf thickness (Lthick), leaf toughness (Ltough), and wood specific gravity (WSG). Blue and green colours denote traits pertaining to water relations and carbon use respectively. Circles show posterior medians of standardised coefficients, and lines indicate 95% highest posterior density intervals [HPDI]. Model covariates were considered to have a clear effect when their slope coefficients 95%-HPDIs did not encompass zero. Filled circles indicate clear negative or positive effects (i.e. slope coefficient 95%-HPDIs not encompassing zero) and empty circles indicate no effects. Positive parameter estimates for β_{1-3} indicate faster growth with increasing values of the model covariate, while negative parameter estimates indicate slower growth with increasing model covariate. Positive β_5 values indicate a buffering effect of NCI on climate, while negative β_5 values indicate an accentuating effect of NCI on climate. For details on parameter estimates see: Table S2.

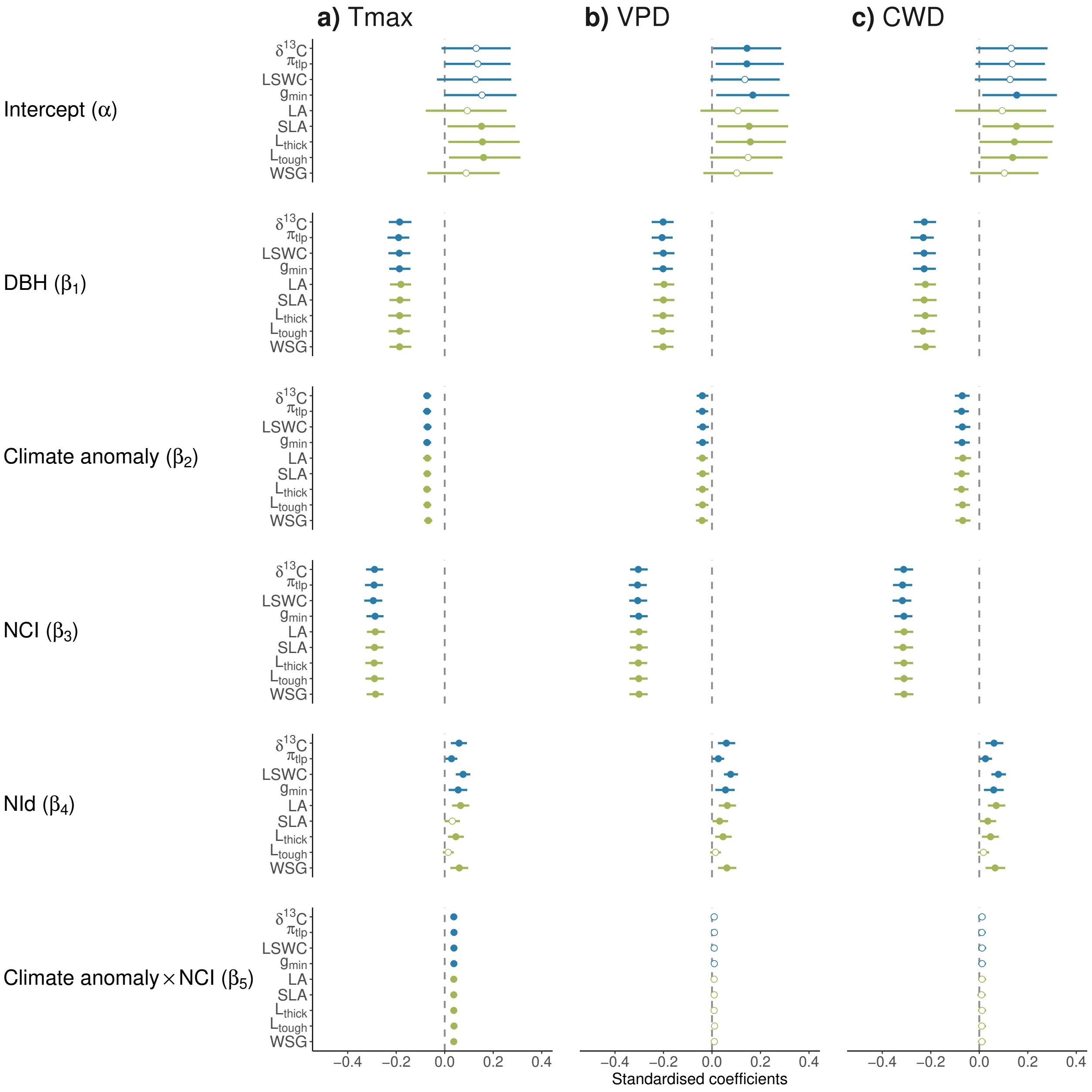

Figure **S5.** Community-level parameter estimates from the 27 NId models showing intrinsic AGR (Intercept, α) and community level AGR response to tree size (DBH, β_1), climate anomaly (β_2), neutral neighbourhood crowding (NCI, β_3), the dissimilarity neighbourhood index (NId, β_4) and the interactions between climate anomaly and neutral neighbourhood crowding (Climate anomaly×NCI, β_5). Parameter estimates of the response to NId (β_4) and the interactions between climate anomaly and NId (β_6) are presented in the main text (Fig. 2 and 3. Standardised coefficients are shown separately for the three climate models: a) Tmax, b) VPD and c) CWD models and each of the nine trait models: carbon $\delta^{13}C$ isotope composition, water potential at turgor loss point (π_{tlp}), leaf saturated water content (LSWC), minimum conductance (g_{min}), leaf area (LA), specific leaf area (SLA), leaf thickness (Lthick), leaf toughness (Ltough), and wood specific gravity (WSG). Blue and green colours denote traits pertaining to water relations and carbon use respectively. Circles show posterior medians of standardised coefficients, and lines indicate 95% highest posterior density intervals [HPDI]. Model covariates were considered to have a clear effect when their slope coefficients 95%-HPDIs did not encompass zero. Filled circles indicate clear negative or positive effects (i.e. slope coefficient 95%-HPDIs not encompassing zero) and empty circles indicate no effects. Positive parameter estimates for β_{1-3} indicate faster growth with increasing values of the model covariate, while negative parameter estimates indicate slower growth with increasing model covariate. Positive β_5 values indicate a buffering effect of NCI on climate, while negative β_5 values indicate an accentuating effect of NCI on climate. For details on parameter estimates see: Table S2.

Supplementary Methods

Methods S1: Corrections of tree inventory data

To ensure the quality of the inventory data set and the completeness of the neighbourhood several corrections have been made. We excluded growth measurements of focal individuals calculated from estimated but not measured DBH as well as abnormal tree growth values (absolute decreases over 2 cm and annual increases over 5 cm per year, thresholds for abnormal changes in DBH were based on expert knowledge for the site). We further discarded individuals with DBH estimations due to buttresses, human induced damage (e.g. through logging treatments) as well as missing or uncertain botanical determination from the focal tree data set. For neighborhood trees with missing or abnormal circumference, we proceeded as follows: we estimated tree circumference from the individual's growth trajectory for census years in which trees were not observed or falsely classified as dead, thus reappearing as alive at a later census. We further corrected abnormal annual increases ($>$ 5 cm) and absolute decreases ($<$ 2 cm) in DBH by aligning the concerned DBH measurement to the overall growth trajectory of the individual (Page *et al.*, 2021).

Methods S2: Gapfilling of missing species information

The vernabota R package (Derroire *et al.*, 2022) gapfills missing botanical species names using vernacular names by combining prior knowledge and observations from our site in a Multinomial-Dirichlet scheme to create a matrix of probability of association between vernacular and botanical names (Aubry-Kientz *et al.*, 2013). Tests performed on fully determined individuals allowed to select the best settings for the gapfilling and showed that the correct botanical name was retrieved in 81,42 [73,18 - 91,45] % (median, [range]) of the cases (for more information on the overall method see Derroire *et al.* 2022: `https://ecofog.github.io/vernabota/`).

Methods S3: Calculation of climate anomalies

To calculate mean standardised climate anomalies (CA) over each of the two-year census intervals, we first calculated the 30-year baseline (historical mean, $\mu_{m,baseline}$) and standard deviation ($\sigma_{m,baseline}$) separately for each month of the year for the 1991-2021 period. For each month (1-12) m at year t, we then calculated deviations of the mean monthly climate variable from its long-term month specific baseline ($\mu_{m,baseline}$) and divided these monthly deviations by their month specific standard deviation ($\sigma_{m,baseline}$). To get standardised mean anomalies (CA_t) for each of the two-year census intervals, we averaged these monthly values over the 24 months prior to each census t, i.e. over year $y = t - 1$ and year $y = t$ (a year being considered from July to July (Aubry-Kientz *et al.*, 2015;

Bauman *et al.*, 2022; Nemetschek *et al.*, 2024; Rifai *et al.*, 2018):

$$CA_t = \frac{1}{24} \sum_{y=t-1}^{t} \sum_{m=1}^{12} \frac{\mu_{m,y} - \mu_{m,baseline}}{\sigma_{m,baseline}} \quad \text{(eqn S1)}$$

The resulting standardised monthly climate anomaly terms are therefore expressed in units of standard deviation from their long term monthly mean.

Methods S4: Additional information on neighbourhood indices

We chose a 10 m radius, as it relates to other recent studies in tropical forests (Fortunel *et al.*, 2018; Lasky *et al.*, 2014; Uriarte *et al.*, 2016) and it has been shown to be sufficient to capture neighbourhood effects while keeping edge effects low (Zambrano *et al.*, 2020). To avoid incomplete neighbourhoods, all focal tree individuals closer than 10 m to the plot edge were excluded.

NIh and NId require trait information for all neighbours within the neighbourhood. Although LA and carbon use traits were available for a greater number of species than water-related traits, we only kept measured species trait information for focal trees and neighbouring species for which we had complete trait information for all nine traits (89 species), to keep the number of species with measured trait information constant between trait models. This eased the interpretation on the importance of trait dissimilarities and hierarchies between trait models, particularly between water-related and carbon use traits. To reduce the influence of missing species trait information on neighbourhood effect estimates, we only modelled (see Models section) growth of focal trees belonging to the 89 species with measured trait values and for which at least 75% of the NCI belonged to neighbouring species with measured traits values (25,686 individuals, 260,058 growth measurements). We gap-filled trait information for all 580 remaining species representing 21.48% of all neighbouring stems, using the year and plot specific community-weighted mean, to account for differences in community composition across plots and years.

Methods S5: Transformation of response variable and model covariates

To reduce the influence of outliers and heteroscedasticity of the growth data, and to represent the multiplicative effects of covariates, we modelled the natural logarithm of absolute growth rates $log(AGR)$ (Fortunel *et al.*, 2018; Hérault *et al.*, 2011; Kunstler *et al.*, 2016). To deal with negative and zero growth before taking the logarithm, we added a constant C_{AGR} to the observed growth value AGR ($C_{AGR} = |AGR_{min}| + |AGR_{min}|/10$), where AGR_{min} is the minimum growth value in our growth dataset (Bauman *et al.*, 2022). As we assumed tree growth to have a non-linear relationship with DBH (Canham *et al.*, 2004), NCI (Fortunel *et al.*, 2016), NIh and NId, we log-transformed these four model covariates prior to standardisation (Fortunel *et al.*, 2018; Kunstler *et al.*, 2016). As NIh can also be negative (i.e. the neighbourhood has overall higher trait values relative to the focal tree), we added a constant C_{NIh} to NIh ($C_{NIh} = |NIh_{min}| + 0.1$), before taking the logarithm, where NIh_{min} is the minimum NIh value

in our data set. To allow for direct comparison of parameter estimates within and between models and ease the assignment of plausible weakly-informative prior to the parameters (McElreath, 2020), $log(AGR)$ and all covariates were standardised to mean zero and unit standard deviation, except for climate anomalies (Bauman *et al.*, 2022; Nemetschek *et al.*, 2024). As our focal species cover a wide range of mean tree sizes, we standardised DBH to mean zero and unit standard deviation within species, to prevent confounding species differences in growth response to tree size with inter-specific variation in mean DBH (Fortunel *et al.*, 2018).

Methods S6: Full model equation

For each individual i of species s between censuses $t-2$ and t, we modeled tree growth as:

$$\log(AGR_{i,s,t,p}) \sim \mathcal{N}(\mu_{i,s,t,p}, \sigma^2) \tag{eqn S2a}$$

$$\begin{aligned}
\mu_{i,s,t,p} &= \alpha_s + \beta_{1s} \times \log(DBH_{i,t-2}) + \beta_{2s} \times CA_t \\
&+ \beta_{3s} \times \log(NCI_{i,t-2}) + \beta_{4s} \times \log(NI_{i,t-2}) \\
&+ \beta_{5s} \times CA_t \times \log(NCI_{i,t-2}) + \beta_{6s} \times CA_t \times \log(NI_{i,t-2}) \\
&+ \gamma_p + \epsilon_i
\end{aligned} \tag{eqn S2b}$$

$$\begin{pmatrix} \alpha_s \\ \beta_{1s} \\ \vdots \\ \beta_{6s} \end{pmatrix} \sim MVNormal \left[\begin{pmatrix} \alpha \\ \beta_1 \\ \vdots \\ \beta_6 \end{pmatrix}, S \right] \tag{eqn S2c}$$

$$S = \begin{pmatrix} \sigma_\alpha & 0 & 0 & 0 \\ 0 & \sigma_{\beta_1} & 0 & 0 \\ \vdots & \vdots & \ddots & \vdots \\ 0 & 0 & 0 & \sigma_{\beta_6} \end{pmatrix} \times R \times \begin{pmatrix} \sigma_\alpha & 0 & 0 & 0 \\ 0 & \sigma_{\beta_1} & 0 & 0 \\ \vdots & \vdots & \ddots & \vdots \\ 0 & 0 & 0 & \sigma_{\beta_6} \end{pmatrix} \tag{eqn S2d}$$

$$R = \begin{pmatrix} 1 & \rho_{\alpha,\beta_1} & \rho_{\alpha,\beta_{\dots}} & \rho_{\alpha,\beta_6} \\ \rho_{\alpha,\beta_1} & 1 & \rho_{\beta_1,\beta_{\dots}} & \rho_{\beta_1,\beta_6} \\ \vdots & \vdots & \vdots & \vdots \\ \rho_{\alpha,\beta_6} & \rho_{\beta_1,\beta_6} & \rho_{\beta_{\dots},\beta_6} & 1 \end{pmatrix} \tag{eqn S2e}$$

$$\begin{aligned}
\gamma_p &\sim \mathcal{N}(0, \sigma_\gamma^2) \\
\epsilon_i &\sim \mathcal{N}(0, \sigma_\epsilon^2) \\
\alpha, \beta_{1-6} &\sim \mathcal{N}(0, 0.5) \\
\sigma_\alpha^2, \sigma_{\beta_{1-6}}^2, \sigma_\gamma^2, \sigma_\epsilon^2, \sigma^2 &\sim \exp(1) \\
R &\sim LKJcorr(2)
\end{aligned} \tag{eqn S2f}$$

where α_s represents the species-level intrinsic growth and β_{1s}, β_{2s}, β_{3s}, β_{4s}, β_{5s} and β_{6s} represent species-level

growth responses to tree size (DBH), climate anomalies (CA), neighbourhood crowding (NCI), the neighbourhood index (NI) either capturing trait hierarchies (NIh) or dissimilarities (NId), the interactive effect of climate anomalies and neighbourhood crowding ($CA \times NCI$) and the interactive effect of climate anomalies and the neighbourhood index considered ($CA \times NI$) (eqn S2a). Negative values of α_s indicate species whose growth is lower and positive values indicate species whose growth is higher than the average AGR across all species. Negative values of β_{1-4s} indicate decreases in growth with increasing model covariate. The interpretation of β_{5s} and β_{6s} depends on the respective directions of β_{2s} and β_{3-4s}. If the sign of β_{5-6s} is the same as the signs of β_{2s} and β_{3-4s}, it means that the interaction between neighbourhood indices and climate can accentuate their separate effects. Conversely, if the sign of β_{5-6s} is opposite from both signs of β_{2s} and β_{3-4s}, the interaction between climate anomalies and neighbourhood indices can attenuate their separate effects.

Species intrinsic growth α_s and growth response to covariates β_{1-4s} for the s species were modelled as in eqn S2c, where α represents intrinsic growth rate and β_{1-6} the overall effect of covariates on growth across all species. To assess correlations (ρ) among species-level intrinsic growth α_s and growth response to model covariates β_{1-6s}, we fitted a matrix of correlation coefficients among all pairs of species-level parameters. The variance-covariance matrix S was constructed as shown in eqn S2d, where R is a matrix of correlation coefficients ρ among all pairs of species-level parameters (eqn S2e). Varying intercepts for the p plots γ_p and the i individuals ϵ_i were modelled as given in eqn S2f with a mean centred on 0 (i.e. mean intercept across all plots and individuals respectively). We specified weakly informative priors which are given in eqn S2f.

Methods S7: Information on model stability

Model estimates for community level intercept, the effect of focal tree DBH and separate effect of NCI on growth were consistent across all neighbourhood index-climate-trait model combinations (Table S2, Fig. S4 and S5) and with findings from our previous study (Nemetschek *et al.*, 2024). As these community-level parameters should be independent from the chosen climate variable and functional trait, this indicates that our selected modelling approach is highly stable and leads to reliable results. Further highlighting the stability of parameter estimates from our models, separate effects of neighbourhood indices including trait hierarchies (NIh) and dissimilarities (NId) were consistent across climate models and climate effects were stable across neighbourhood index-trait model combinations (Fig. S4 and S5).

Methods S8: Spatial autocorrelation check

To ensure that our results were not driven by spatial autocorrelation, we checked for the absence of strong correlations among model residuals. To do so we extracted the model residuals for each of 54 models using the residuals() function of the brms R package (Bürkner, 2017). Using the corrlog() function of the ncf R package (Bjornstad, 2022) we then calculated a spatial correlogram, using 99 permutations, for the residuals of each of the 54 models

for every possible plot - census year combination (15 plots x 15 census years x 54 models = 12150 plot-year-model combinations). The correlogram estimates the spatial dependence of all pairs of individual residuals belonging to a given distance class (2 m distance classes starting from 0 m to 120 m, resulting into 61 distance classes). This follows the rule of thumb for testing spatial autocorrelation for one-third of the diagonal of the spatial extent of the data (rectangle of 250 m in our case). We found that the residuals were not strongly correlated and are therefore confident that there is no bias in our approach due to pseudo-replication (see Table S6).

References

Aubry-Kientz, M., Hérault, B., Ayotte-Trépanier, C., Baraloto, C. & Rossi, V. (2013). Toward Trait-Based Mortality Models for Tropical Forests. *PLoS ONE*, 8, e63678.

Aubry-Kientz, M., Rossi, V., Wagner, F. & Hérault, B. (2015). Identifying climatic drivers of tropical forest dynamics. *Biogeosciences*, 12, 5583–5596.

Bauman, D., Fortunel, C., Cernusak, L.A., Bentley, L.P., McMahon, S.M., Rifai, S.W., Aguirre-Gutiérrez, J., Oliveras, I., Bradford, M., Laurance, S.G.W., Delhaye, G., Hutchinson, M.F., Dempsey, R., McNellis, B.E., Santos-Andrade, P.E., Ninantay-Rivera, H.R., Chambi Paucar, J.R., Phillips, O.L. & Malhi, Y. (2022). Tropical tree growth sensitivity to climate is driven by species intrinsic growth rate and leaf traits. *Global Change Biology*, 28, 1414–1432.

Bjornstad, O.N. (2022). *ncf: Spatial Covariance Functions*.

Bürkner, P.C. (2017). brms: An R Package for Bayesian Multilevel Models Using Stan. *Journal of Statistical Software*, 80, 1–28.

Canham, C.D., LePage, P.T. & Coates, K.D. (2004). A neighborhood analysis of canopy tree competition: effects of shading versus crowding. *Canadian Journal of Forest Research*, 34, 778–787.

Derroire, G., Aubry-Kientz, M., Mirabel, A., Marcon, E. & Bruno, H. (2022). vernabota: Association between vernacular and botanical names for Guyafor data.

Fortunel, C., Lasky, J.R., Uriarte, M., Valencia, R., Wright, S.J., Garwood, N.C. & Kraft, N. (2018). Topography and neighborhood crowding can interact to shape species growth and distribution in a diverse Amazonian forest. *Ecology*, 99, 2272–2283.

Fortunel, C., Valencia, R., Wright, S.J., Garwood, N.C. & Kraft, N.J. (2016). Functional trait differences influence neighbourhood interactions in a hyperdiverse Amazonian forest. *Ecology letters*, 19, 1062–1070.

Hérault, B., Bachelot, B., Poorter, L., Rossi, V., Bongers, F., Chave, J., Paine, C.E.T., Wagner, F. & Baraloto, C. (2011). Functional traits shape ontogenetic growth trajectories of rain forest tree species. *Journal of Ecology*, 99, 1431–1440.

Kunstler, G., Falster, D., Coomes, D.A., Hui, F., Kooyman, R.M., Laughlin, D.C., Poorter, L., Vanderwel, M., Vieilledent, G., Wright, S.J., Aiba, M., Baraloto, C., Caspersen, J., Cornelissen, J.H.C., Gourlet-Fleury, S., Hanewinkel, M., Herault, B., Kattge, J., Kurokawa, H., Onoda, Y., Peñuelas, J., Poorter, H., Uriarte, M.M., Richardson, S., Ruiz-Benito, P., Sun, I.F., Ståhl, G., Swenson, N.G., Thompson, J., Westerlund, B., Wirth, C.,

Zavala, M.A., Zeng, H., Zimmerman, J.K., Zimmermann, N.E. & Westoby, M. (2016). Plant functional traits have globally consistent effects on competition. *Nature*, 529, 204–207.

Lasky, J.R., Uriarte, M., Boukili, V.K. & Chazdon, R.L. (2014). Trait-mediated assembly processes predict successional changes in community diversity of tropical forests. *Proceedings of the National Academy of Sciences*, 111, 5616–5621.

McElreath, R. (2020). *Statistical rethinking: A Bayesian course with examples in R and Stan.* CRC Press.

Nemetschek, D., Derroire, G., Marcon, E., Aubry-Kientz, M., Auer, J., Badouard, V., Baraloto, C., Bauman, D., Le Blaye, Q., Boisseaux, M., Bonal, D., Coste, S., Dardevet, E., Heuret, P., Hietz, P., Levionnois, S., Maréchaux, I., McMahon, S.M., Stahl, C., Vleminckx, J., Wanek, W., Ziegler, C. & Fortunel, C. (2024). Climate anomalies and neighbourhood crowding interact in shaping tree growth in old-growth and selectively logged tropical forests. *Journal of Ecology*, 0.

Page, N., Derroire, G., Hérault, B., Marcon, E., Guedj, E., Jaouen, G. & Piponiot, C. (2021). *ForestData: Post-inventory processing of forest plot data.*

Rifai, S.W., Girardin, C.A., Berenguer, E., Del Aguila-Pasquel, J., Dahlsjö, C.A., Doughty, C.E., Jeffery, K.J., Moore, S., Oliveras, I., Riutta, T., Rowland, L.M., Murakami, A.A., Addo-Danso, S.D., Brando, P., Burton, C., Ondo, F.E., Duah-Gyamfi, A., Amézquita, F.F., Freitag, R., Pacha, F.H., Huasco, W.H., Ibrahim, F., Mbou, A.T., Mihindou, V.M., Peixoto, K.S., Rocha, W., Rossi, L.C., Seixas, M., Silva-Espejo, J.E., Abernethy, K.A., Adu-Bredu, S., Barlow, J., Da Costa, A.C., Marimon, B.S., Marimon-Junior, B.H., Meir, P., Metcalfe, D.B., Phillips, O.L., White, L.J. & Malhi, Y. (2018). ENSO Drives interannual variation of forest woody growth across the tropics. *Philosophical Transactions of the Royal Society B: Biological Sciences*, 373, 20170410.

Uriarte, M., Lasky, J.R., Boukili, V.K. & Chazdon, R.L. (2016). A trait-mediated, neighbourhood approach to quantify climate impacts on successional dynamics of tropical rainforests. *Functional Ecology*, 30, 157–167.

Zambrano, J., Beckman, N.G., Marchand, P., Thompson, J., Uriarte, M., Zimmerman, J.K., Umaña, M.N. & Swenson, N.G. (2020). The scale dependency of trait-based tree neighborhood models. *Journal of Vegetation Science*, 31, 581–593.
